# Terrestrial support of lake food webs: Synthesis reveals controls over cross-ecosystem resource use

**DOI:** 10.1126/sciadv.1601765

**Published:** 2017-03-22

**Authors:** Andrew J. Tanentzap, Brian W. Kielstra, Grace M. Wilkinson, Martin Berggren, Nicola Craig, Paul A. del Giorgio, Jonathan Grey, John M. Gunn, Stuart E. Jones, Jan Karlsson, Christopher T. Solomon, Michael L. Pace

**Affiliations:** 1 Ecosystems and Global Change Group, Department of Plant Sciences, University of Cambridge, Cambridge CB2 3EA, U.K.; 2 Department of Forest and Conservation Sciences, University of British Columbia, Vancouver, British Columbia V6T 1Z4, Canada.; 3 Department of Environmental Sciences, University of Virginia, Charlottesville, VA 22904, USA.; 4 Department of Physical Geography and Ecosystem Science, Lund University, S-223 62 Lund, Sweden.; 5 Department of Natural Resource Sciences, McGill University, Sainte Anne de Bellevue, Quebec H9X 3V9, Canada.; 6 Département des Sciences Biologiques, Université du Québec à Montréal, Montréal, Quebec H3C 3P8, Canada.; 7 Lancaster Environment Centre, Lancaster University, Lancaster LA1 4YQ, U.K.; 8 The Wild Trout Trust, PO Box 120, Waterlooville PO8 0WZ, U.K.; 9 Vale Living with Lakes Centre, Laurentian University, Sudbury, Ontario P3E 2C6, Canada.; 10 Department of Biological Sciences, University of Notre Dame, Notre Dame, IN 46556, USA.; 11 Climate Impacts Research Centre, Department of Ecology and Environmental Science, Umeå University, 90187 Umeå, Sweden.; 12 Cary Institute of Ecosystem Studies, Millbrook, NY 12545, USA.

**Keywords:** allochthony, food webs, land-water linkages, organic carbon

## Abstract

Widespread evidence that organic matter exported from terrestrial into aquatic ecosystems supports recipient food webs remains controversial. A pressing question is not only whether high terrestrial support is possible but also what the general conditions are under which it arises. We assemble the largest data set, to date, of the isotopic composition (δ^2^H, δ^13^C, and δ^15^N) of lake zooplankton and the resources at the base of their associated food webs. In total, our data set spans 559 observations across 147 lakes from the boreal to subtropics. By predicting terrestrial resource support from within-lake and catchment-level characteristics, we found that half of all consumer observations that is, the median were composed of at least 42% terrestrially derived material. In general, terrestrial support of zooplankton was greatest in lakes with large physical and hydrological connections to catchments that were rich in aboveground and belowground organic matter. However, some consumers responded less strongly to terrestrial resources where within-lake production was elevated. Our study shows that multiple mechanisms drive widespread cross-ecosystem support of aquatic consumers across Northern Hemisphere lakes and suggests that changes in terrestrial landscapes will influence ecosystem processes well beyond their boundaries.

## Teaser

There is widespread evidence that aquatic consumers use terrestrial resources depending on the features of surrounding catchments.

## INTRODUCTION

Ecosystems are linked across landscapes by the flow of energy and nutrients (*1*). This has long been evident at the scale of catchments, wherein terrestrial organic matter (t-OM) is collected by hydrological flow and funneled into receiving waterways at lower elevation. Aquatic organisms are consequently able to use material produced outside the boundaries of their habitat—a process known as allochthony—to support their metabolic demands (*2*). Accumulating evidence now suggests that the use of terrestrially derived resources can be as high as 40 to 94% in some lake food webs (*3*–*13*). Cross-ecosystem resources therefore play an important role in supporting ecosystem functioning and the delivery of key ecosystem services, such as fish production (*9*, *14*). Yet, it remains unclear as to how reliance on these resources will change with continued human degradation or, alternatively, restoration of the planet’s landscapes.

Generalizing how cross-ecosystem resources support lentic consumers in particular has been controversial (*6*, *15*, *16*). In addition to grazing on phytoplankton and microbial organisms that decompose detritus generated within aquatic ecosystems, zooplankton ingest microbes that metabolize t-OM, and they directly uptake t-OM through feeding (*17*–*19*). These t-OM sources can sustain consumer growth and reproduction as long as some high-quality resources, such as green algae, are present (*15*, *19*). However, t-OM is generally a poor-quality resource. It lacks essential fatty acids and macronutrients available from algae (*15*, *20*). Rather, t-OM likely supplements existing within-lake resources as it becomes increasingly available (*9*) and/or the latter are limited [for example, seasonally (*3*, *21*)], elevating allochthony without necessarily increasing consumer production (*16*, *22*–*24*).

The low levels of allochthony (<20%) observed in some studies of lake food webs have also cast doubt on the importance of cross-ecosystem resources in supporting consumer biomass (*25*–*27*). This leaves considerable variation to be explained among studies, both within and across geographic regions (*8*, *21*, *28*, *29*). Nonetheless, theory and meta-analyses of consumer abundances can be used to predict that allochthonous resource fluxes will be most used when (i) receiving food webs have low productivity or relatively few resources, (ii) the delivery potential of donor habitats is relatively large, and/or (iii) consumers have weak preferences for autochthonous resources (*30*–*32*). The relative importance of spatial energy flows will also depend on temporal variation in food web structure, such as those arising from seasonal changes in primary production (*33*). Therefore, previous disagreements over the importance of terrestrial support may have arisen because lakes differ in their productivity either spatially and/or temporally, are surrounded by different land uses, and have different zooplankton assemblages. Empirically testing these general predictions across diverse habitats can help reconcile contrasting findings.

Finally, controversy has arisen over the methods used to measure allochthony, which are primarily based on stable isotope mixing models (*15*, *34*, *35*). Rigorous simulation approaches are now needed to understand how mixing models perform under different empirical conditions and identify potential sources of bias, such as in end-member determination. Taken with concerns around the nutritional quality of t-OM and the large variability in observed allochthony, the general conditions under which terrestrial resources are important to lake food webs remain to be identified. Accordingly, such an analysis can also reveal the conditions under which the use of autochthonous resources varies.

Here, we test how within-lake processes and catchment-level characteristics jointly influence the use of terrestrial and within-lake resources in aquatic food webs, thereby explaining the large variation in allochthony reported to date. Because our study sites were a nonrandom collection of lakes for which terrestrial resources were likely more important, on average, than elsewhere, we focused on understanding when, and for which consumers, allochthony was high in our subset of global lake types. We did so by simultaneously testing the following five mutually inclusive hypotheses around cross-ecosystem resources and comparing their relative support:

(1) Favorable resources hypothesis: Allochthony decreases when more high-quality resources (that is, algae) are produced (*5*, *21*).

(2) Catchment deposition hypothesis: Allochthony increases as more t-OM is exported from the surrounding catchment. A greater quantity and reactivity of t-OM can be made available for consumption by consumers at the base of aquatic food webs as the coverage and density of labile vegetation and soil carbon in the surrounding catchment increase (*9*, *36*). A larger land-water interface can also increase the geomorphic potential of catchments to deliver t-OM into receiving food webs (*14*, *37*).

(3) Algal subtraction hypothesis: Allochthony increases with the availability of t-OM, where algal production becomes limited by shading more than it benefits from the nutrients associated with t-OM (*38*).

(4) Consumer preference hypothesis: Allochthony increases in consumers (such as Cladocera) that are relatively effective grazers of bacterial decomposers and t-OM as these resources become increasingly available (*28*, *39*, *40*).

(5) Seasonality hypothesis: Allochthony increases outside of the summer growing season when plant litter production peaks and/or within-lake production is negligible (*3*, *40*, *41*).

Our analysis is the first large-scale effort that explicitly links resource use by aquatic organisms to catchment characteristics, such as land cover. We collated stable isotope (δ^2^H, δ^13^C, and δ^15^N) measurements taken year-round for 559 observations of pelagic consumers across 147 lakes in many of the planet’s freshwater hot spots. Using a uniform approach to delineating study catchments, we also assembled an extensive database on surrounding land use, geomorphology, and water chemistry derived from satellite and field data. We then estimated terrestrial resource use (φ_T_) in an isotopic mixing model by relating φ_T_ to within-lake and catchment characteristics using structural equation modeling (SEM) (methods S1 and S2). SEM allows us to test explicitly the strength and direction of five of the major hypotheses about allochthony. Previously, we have shown that bias in the mixing model approach is minimal (*7*, *9*, *42*), and we expand on these analyses to show that it is relatively insensitive to both the range of isotopic values observed in our data set and missing data sources.

## RESULTS

### Isotopic mixing model performance

**Fig. 1:**
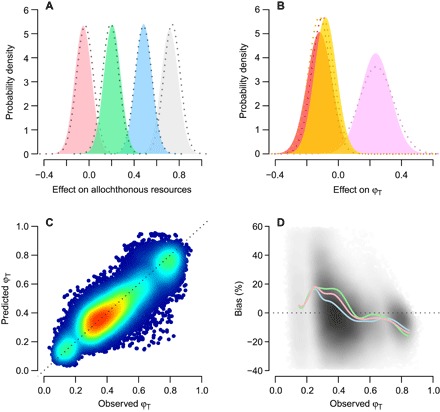
Model recovers known parameters across 100 simulated data sets that replicate our empirical observations. Mean posterior distributions of the effects of (**A**) DOC (gray), normalized difference vegetation index (NDVI) (pink), ratio of lake perimeter to area (blue), and area of woody vegetation per meter shoreline (green) on availability of allochthonous resources and (**B**) allochthonous resources (purple), lake chlorophyll *a* (red), and an allochthonous resources–chlorophyll *a* interaction (orange) on terrestrial resource use (ϕ_T_); dashed lines are known prior distributions. (**C**) Mean predicted ϕ_T_ versus observed (that is, known) ϕ_T_ for 559 consumer observations in each of the 100 simulations. Warmer colors indicate greater concentration of points (total *n* = 55,900). (**D**) Percent bias in mean predicted ϕ_T_ values. Darker shading indicates greater concentration of points. Lines are splines fitted through observations on one (δ^2^H only; pink), two (δ^13^C-δ^15^N; green), or three (δ^13^C-δ^15^N-δ^2^H; blue) isotopes.

The isotopic mixing model that we developed had sufficient power to test our focal hypotheses, because it strongly recovered known values of φ_T_ and its response to extrinsic factors. We simulated data sets that replicated our empirical observations with known effect sizes and found that posterior distributions for the effects of lake- and catchment-level characteristics on φφ_T_ averaged across 100 simulations were tightly centered on their “true” values (dotted lines versus polygons in Fig. 1, A and B). φ_T_ was also recovered with relatively high precision and accuracy (Fig. 1C). Although there was a tendency to overestimate moderate values of φ_T_ (ca. 0.20 to 0.40) with a relative bias, on average, of up to 18% (absolute deviation in φ_T_ of 7%), most relative bias was small and ranged between −10 and 10% (Fig. 1D). Data for δ^2^H isotopes further reduced this bias (Fig. 1D), because it most strongly differentiated between our two basal food sources of terrestrial plants and pelagic phytoplankton (fig. S1).

We also considered whether our results could be biased by the different basal food resources and isotopes that we studied. Isotopic signatures of terrestrial resources loaded into food webs, which were measured from fresh or senesced leaves of the dominant plants or soil OM in surrounding catchments, varied little as compared to those of within-lake resources (fig. S1). Pelagic phytoplankton, for which isotopic signatures were directly measured for *n* = 333 consumer observations and estimated in another *n* = 226 from their photosynthetic δ^2^H discrimination, varied much more in δ^13^C and δ^15^N than terrestrial resources, with no clear difference between the measured and estimated values (fig. S1). Nonetheless, the variation in the observed resources had little influence on our results. We found that bias in both φ_T_ and its response to lake- and catchment-level characteristics was unchanged when we increased the uncertainty in the allochthonous and autochthonous resources that were input into the mixing model (light and dark green lines no different from the gray box in fig. S2). Additional simulations showed that all focal parameters were relatively insensitive to increased uncertainty in other sources, such as the isotope measurements themselves (fig. S2), biased prior information about consumer physiology (fig. S3), and potentially missing resources that would bias determination of the within-lake resources, such as methane-oxidizing bacteria (MOB) (fig. S4).

### Mechanisms underlying resource use

**Fig. 2:**
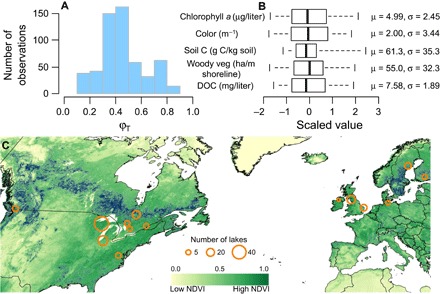
Terrestrial resource (ϕ_T_) use by lake zooplankton. (**A**) Mean posterior estimates of ϕ_T_ for each of the 559 consumer observations. (**B**) Scaled distributions of key catchment characteristics and unscaled means and SDs. (**C**) Focal lake regions (*n* = 14) superimposed on water bodies at a resolution of 1 km and a proxy of vegetation density (NDVI) at a resolution of 0.1° in September 2015 (NASA Earth Observations data repository, http://neo.sci.gsfc.nasa.gov/).

Given our validated model, we found that φ_T_ estimated for aquatic consumers based on empirical δ^2^H, δ^13^C, and δ^15^N data varied greatly across gradients of water quality and catchment characteristics (Fig. 2; see data file S1 for site summaries). Mean [95% credible interval (CI)] estimates of consumer biomass derived from terrestrial resources ranged between 11% (3 to 21%) and 83% (72 to 92%) across 147 lakes, with a median of 42% (Fig. 2). The model generating these estimates fitted the empirical data very well (Bayesian *R*^2^ ranging from 0.64 to 0.99 across observations in the one-, two-, and three-isotope models; fig. S5). As the estimates were sampled using Bayesian inference, we also generated a posterior distribution of φ_T_ for each of our 559 consumer observations. The resulting distributions were always peaked with almost all coefficients of variation <0.4 (fig. S6), emphasizing low uncertainty in our predictions.

**Fig. 3:**
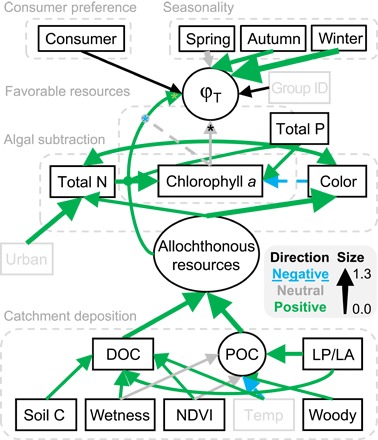
Modeled network of factors influencing terrestrial resource use (ϕ_T_) by aquatic consumers across 147 lakes. Arrows point at modeled variables, with mean effects of one variable on another proportional to standardized effect size (see legend). Lines ending in circles are interactions. The asterisk symbol (*) indicates random variation among consumers, with colors showing direction of significant effects. Black lines are intercepts with no “effect direction,” ellipses are unobserved (that is, latent) variables, and gray boxes are covariates included to explain the connections between modeled variables and predictors of interest better. Five mechanisms explaining variation in ϕ_T_ are associated with broken boxes. NDVI, vegetation density; temp, mean monthly temperature of warmest quarter; woody, area of woody vegetation in catchment per meter shoreline; LP/LA, ratio of lake perimeter to lake area; group ID, research group that collected the data (such as to account for variation in sampling). Bayesian *R*^2^ for consumers with one (δ^2^H only), two (δ^13^C-δ^15^N), or three (δ^13^C-δ^15^N-δ^2^H) observed isotopes were 0.64, 0.98, and 0.99, respectively (fig. S5).

**Fig. 4:**
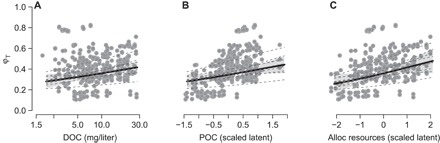
ϕ_T_ increases with t-OM. Specifically, ϕT increased with the estimated availability of DOC (**A**), POC (**B**), and their summed contribution toward allochthonous (alloc) resources (**C**). Points are mean estimated ϕ_T_ values for each of the 409 consumer observations with corresponding water chemistry measurements. The solid line denotes the mean increase across all consumers at mean levels of all other water chemistry variables, with the shaded polygon denoting 95% CI and dotted lines denoting consumer-specific responses.

**Fig. 5:**
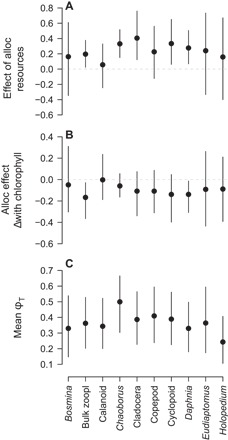
Consumer-specific variation in ϕ_T_. Means ± 95% CIs plotted for the effect of allochthonous (alloc) resources on ϕ_T_ (**A**), the change in effect of allochthonous resources on ϕ_T_ with increasing lake water chlorophyll *a* (**B**), and ϕ_T_ at mean water chemistry levels across sites (**C**). zoopl, zooplankton.

For the first time, we could link the large variation in φ_T_ found in Fig. 2A and across previous studies to explicit mechanisms that predicted when autochthonous resources versus allochthonous resources would be important. We did so by connecting the distributions of φ_T_ for each consumer observation to within-lake and catchment characteristics and estimating whether the associated 95% CIs excluded 0 (shown as green and blue arrows for positive and negative effects, respectively, in Fig. 3). First, we found that support for the catchment deposition hypothesis operated via both particulate organic carbon (POC) exported from woody vegetation, while accounting for variation in terrestrial litter decomposition because of warmer temperatures, and dissolved organic carbon (DOC) contributed by catchments with dense vegetation cover, rich soil carbon pools, and a high degree of soil wetness (green lines for all connections in Figs. 3 and 4, A and B). Greater quantities of t-OM subsequently elevated allochthonous resources (that is, summed contribution of terrestrially derived DOC and POC), thereby increasing allochthony (Fig. 3). For example, a 30% increase in allochthonous resources over their observed range increased φ_T_ in summer by a relative mean of 7% across all taxa (95% CI, 1 to 14) when other effects were at their mean levels (Fig. 4C). We also found that φφ_T_ increased as lakes were smaller relative to their shoreline, as predicted by the catchment deposition hypothesis (green arrows connecting LP/LA to φ_T_ in Fig. 3). Support for the catchment deposition hypothesis persisted with other indicators of terrestrial influence, especially when we considered lake perimeter in the analyses (method S3). Second, we found that the positive response of φ_T_ to increasing allochthonous resources was reduced by increasing within-lake productivity (that is, interaction with chlorophyll *a*) for *Daphnia* and bulk zooplankton (Fig. 5), as predicted by the favorable resources hypothesis (Fig. 3); however, this hypothesis was not supported across all taxa. Third, the interactive effect was weakened as more allochthonous resources shaded the water column and reduced algal productivity, consistent with the subtraction hypothesis (blue line connecting color to chlorophyll *a* in Fig. 3).

Finally, there were clear differences across consumers and seasons in the use of allochthonous resources, as predicted by the consumer preference and seasonality hypotheses, respectively. Less-selective filter feeders such as *Daphnia*, which also often comprised much of the biomass in the Cladocera and bulk zooplankton categories, had greater φ_T_ as allochthonous resources were increasingly available (95% CIs excluding 0; Fig. 5A) but less so where chlorophyll *a* concentrations were high (Fig. 5B). φ_T_ in *Chaoborus*, which integrate the signals of multiple prey items, also responded positively to the availability of allochthonous resources (Fig. 5A). In contrast, more-selective suspension feeders such as the calanoid copepods, including *Eudiaptomus*, did not have a greater φ_T_ as allochthonous resources became more available (95% CIs overlapping 0; Fig. 5, A and B). There was no difference in φ_T_ across consumers under mean water chemistry conditions (Fig. 5C). We also found that mean levels of allochthony were greatest during autumn, when plant litter production peaks, and winter, when within-lake production is minimized (95% CI for difference from summer: 0.36 to 0.77 and 0.20 to 3.1, respectively; Fig. 3). All other parameter estimates are reported in table S1.

An important benefit of our modeling approach is that it allowed us to compare relative support for different hypotheses. For each hypothesis except that of consumer preference, we calculated the change in φ_T_ with an increase in a focal variable from 1 SD beneath to 1 SD above its mean, while all other variables were fixed at mean levels. This revealed that DOC and the ratio of lake perimeter to lake area (an indicator of t-OM delivery potential) had the strongest cumulative effects in our network of interacting mechanisms (Fig. 3), increasing φ_T_ by 1 to 13% through their effects on the availability of allochthonous resources.

## DISCUSSION

Our analysis across lakes from the boreal to subtropics shows that terrestrial resource use is unequivocally important, accounting for at least 42% of consumer biomass in half of all observations, although high levels of allochthony (for example, >60%) are not a general pattern. Concurrently, we have discovered the conditions that make high allochthony possible, helping to explain the tremendous discrepancy observed across stable isotope studies of lake food webs over the last two decades (*6*–*8*, *16*, *21*, *23*, *25*, *26*, *28*). The lowest mean estimate (11%) of allochthony reported here exceeds that observed by others, possibly because our nonrandom sample of study sites largely lacked clear deepwater and eutrophic lakes where primary production is relatively high (*5*, *25*). Our results also offer general insights to understand the fate of spatial resource fluxes, because we have found that allochthonous resources are used more, as determined using stable isotope tracers, in ecosystems that are unproductive and/or well connected to donor habitats. Predictable changes in allochthony along continuous gradients, such as in hydrological connectivity and ecosystem productivity, support theoretical predictions for when cross-ecosystem resources will be most used (*30*–*32*), but have only been empirically reported to our knowledge in two much more local studies (*9*, *14*).

### Mechanisms underlying allochthony

We found support for the favorable resources and catchment deposition hypotheses. These hypotheses suggest that levels of allochthony in freshwater lakes depend on the quantity of t-OM that is delivered into food webs relative to the amount of internal production. It is therefore unsurprising that marked differences in allochthony have previously been reported across lakes that span gradients of trophic state, morphometry, and catchment characteristics (*8*, *21*, *25*, *28*, *29*). Relative exposure of lakes to their surrounding shorelines was an especially important characteristic that drove support for the catchment deposition hypothesis and highlighted the importance of nearshore processes for t-OM export (*43*). Our results also show that allochthony is promoted by dissolved and particulate t-OM. This finding suggests that both direct ingestion of particulate organic matter (POM) (and its associated biofilms) and bacterial decomposition are key to transferring t-OM into aquatic food webs.

Algal production attenuated the effects of increasing allochthonous resources on terrestrial resource support for some consumers, as expected if it is a higher-quality and more preferred resource (*15*, *20*), but this effect was sensitive to shading of the water column, as predicted by the algal subtraction hypothesis. These responses are likely to reflect shifts in the availability of phytoplankton across depth zones as water clarity changes. In deep clear lakes, few of which we studied here, phytoplankton may support most of the zooplankton biomass (*25*). As t-OM increases, reduced light penetration and shallower thermoclines will constrain metalimnetic phytoplankton, decreasing its support of zooplankton (*44*). In contrast, concentrations of DOC comparable to those observed in our data set suggest that t-OM may be sufficient to promote primary productivity in the epilimnion by contributing limiting nutrients without reducing the average amount of radiation reaching phytoplankton cells (*45*). The effects of algal production on allochthony will also vary seasonally (*3*, *40*, *41*), as observed here and predicted by the seasonality hypothesis. Allochthony was specifically lower during spring and summer when algal production was maximized than during autumn leaf fall or winter.

Responses to terrestrial and within-lake resources by the most abundant taxa in our data set were generally consistent with known feeding strategies. For example, calanoids preferentially consume phytoplankton and, thus, do not strongly respond to direct increases in terrestrial resources (*28*, *40*), as we found here. Terrestrial resource use may also change little with small increases in within-lake production if it is already minor (<20%) at low phytoplankton biomass. By contrast, *Daphnia* and cyclopoid copepods benefited from more terrestrial resources because they can graze heterotrophic bacteria associated with dissolved t-OM (*46*, *47*), even during periods of high primary production (*40*). However, only *Daphnia* reduced their use of allochthonous resources with increasing chlorophyll *a*. *Daphnia* are more likely to ingest larger particulate material from leaf fragments or flocculated DOC than calanoids (*28*). Because these materials are poorly assimilated during growth (*15*, *20*), they should be used less often when phytoplankton are available. The lack of an association with chlorophyll *a* in other taxa may be unsurprising if terrestrial resources only sustain growth when supplemented with algae (*15*, *19*). An increasing supply and uptake of algae could thus result in a greater uptake of terrestrial resources without necessarily changing the proportional use of these two resources. Finally, allochthony of the invertebrate predator *Chaoborus* appeared more responsive to terrestrial resources than some of the zooplankton grazers that it preys on, for example, *Bosmina*. This greater responsiveness may have arisen if our samples contained a large number of early instars that proportionally ingest more rotifers, which are enriched in allochthonous resources (*28*), than larger zooplankton, such as *Daphnia* (*47*). *Chaoborus* can also assimilate fewer grazers and more detritivores where they reside in the hypolimnion, such as in lakes with planktivorous fish (*5*). More generally, spatial variation in cross-ecosystem resources should lead to different patterns of allochthony between migratory and more stationary consumers (*1*).

Although we have found support for general mechanisms underlying allochthony, our study sites only partially captured the range of lake physical and chemical characteristics observed globally and within our focal study regions [for example, see the work of Hanson *et al*. and Palmer *et al*. (*48*, *49*)]. Three notable differences emerge from comparisons with global data sets. First, >90% of the world’s lakes have been estimated to be <0.01 km^2^ versus 34% in our data set (*50*). Most of our lakes were slightly larger, with areas between 0.01 and 1 km^2^ (fig. S7). Second, median DOC concentrations in our data set were slightly higher than those in a compilation of 7514 lakes spanning large biogeographic gradients (*51*): 6.9 mg/liter versus 5.7 mg/liter, respectively, suggesting that we may be slightly overestimating the extent of allochthonous inputs and their shading effects (fig. S8). Finally, median chlorophyll *a* concentrations in our data set were nearly 40% lower than satellite-derived estimates in 80,012 lakes (*52*): 4.7 mg/liter versus 7.5 mg/liter, respectively, overrepresenting oligotrophic lakes where allochthony might be higher (fig. S9). The strength of support for some of the mechanisms that we detected might therefore vary in lakes with markedly different characteristics, but the mechanisms themselves remain generalizable in many other cases.

### Improving predictions of cross-ecosystem resource use

We found that estimates of terrestrial resource use were positively biased by an average of up to 18% on a relative basis (ca. 7% on an absolute scale). Extending our analysis across the entire range of potential φ_T_, rather than only the range observed in the empirical data set, revealed that this problem was exacerbated as φ_T_ approached 0 (fig. S10). This was because sampling φ_T_ from a β distribution meant that values could not be <0, thereby limiting negative bias from accruing. Similarly, bias was mostly negative as φ_T_ approached 1 (fig. S10), again because φ_T_ could not be >1. Statistical methods to infer isotopic compositions can do little to account for this given inherent constraints in φ_T_. Caution is therefore needed when evaluating allochthony at extremely low and high values. Estimating cross-ecosystem resource use by enriching distinctions in the isotopic composition of resources [for example, see the work of Pace *et al.* and Wilkinson *et al.* (*4*, *53*)] and measuring additional source-specific biomarkers, such as fatty acids (*27*), may be particularly helpful in these circumstances by reducing underlying isotopic variation and better constraining models to data.

We have also expanded our understanding of the performance and bias of isotopic mixing models (*7*, *9*, *42*), which have been previously critiqued (*15*, *34*, *35*), though without rigorous statistical tests. For end members, we found little difference between isotopic signatures of pelagic phytoplankton that were directly measured versus those inferred from the known discrimination of producers for ^2^H relative to ^1^H in surrounding water, supporting the use of this approach to assigning isotope values (*54*). We similarly found little variation in terrestrial resources despite sometimes measuring either live, recently senesced, or decomposed leaf material. The δ^2^H, δ^13^C, and δ^15^N values of fresh leaves change little as they decay, supporting our grouping of terrestrial material in different states of decomposition (*54*). Bias in the recovery of model parameters was also relatively insensitive to increased uncertainty in the end-member isotope values and physiological parameters used to infer dietary water uptake, as well as moderate levels of missing within-lake resources. Our widespread measurement of δ^2^H for 79% of consumer observations likely helped to reduce bias by differentiating between the isotopic signature of aquatic and terrestrial primary production (*55*). Overall, our analyses highlight the robustness of mixing models and offer opportunities to parameterize them with new types of data, such as fatty acid profiles (*27*).

### New connections in managing land and water resources

Our findings emphasize that better integration is needed across ecosystems in management. We found that consumers rely heavily on terrestrial resources in lakes that are surrounded by relatively long shorelines with dense vegetation and soil carbon stores. This provides empirical support for the conventional wisdom, largely from riparian systems (*56*, *57*), that even small land cover change along shorelines affects lake food webs. Where consumer production benefits from terrestrial resources, our results suggest that reductions in forest cover and soil carbon can reduce the delivery of important services provided by planktonic communities, such as fish production (*9*) or control of algal growth (*58*). Of course, consumers with high terrestrial resource use will not necessarily be more productive. This depends on whether t-OM displaces higher-quality within-lake resources by shading (*16*, *22*, *23*, *38*) or whether it adds more of a suitable food source to the environment (*9*, *19*).

More broadly, our work reveals how terrestrial landscapes influence ecosystem functioning well beyond their boundaries. Much of the north temperate land mass, which stores most of the world’s fresh water, is changing with shifts in climate, natural disturbances, and human activities (*59*). For example, increases in historical fire frequency across the boreal zone are clearing forest twice as quickly as it is being gained (*60*). These changes will affect receiving waters. Our analysis therefore strengthens the need for catchment-level approaches to the management of land and water resources in a changing world.

## MATERIALS AND METHODS

### Sample collection

We sampled 147 lakes on one to five occasions during at least the summer growing season or autumn and, where possible, also during winter and spring. Zooplankton were collected either in bulk (that is, mixed species composition) or at one of nine taxonomic levels: *Bosmina* spp., Calanoida, *Chaoborus* spp., Cladocera, Copepoda, Cyclopoida, *Daphnia* spp., *Eudiaptomus* spp., and *Holopedium* spp. near the deepest point of each lake during the day or night (*Chaoborus* only) using vertical net tows. We also considered an allochthonous and autochthonous basal resource that was available for consumers in each site. These were leaf litter of the dominant terrestrial plants and pelagic phytoplankton, respectively. In some cases, we sampled soil OM or fresh leaves instead of litterfall. These were suitable alternatives because their isotopic ratios have been shown to be indistinguishable from both litterfall and dissolved OM inflowing into lakes (*7*, *54*). Phytoplankton were either collected by net tows during periods of high biomass (*8*, *11*, *21*) or isolated from POM by their specific phospholipid fatty acids (*28*). However, given the difficulty in isolating pure phytoplankton using these two approaches, we inferred their isotopic signatures by sampling environmental water either on its own or with POM for 40% (*n* = 226) of the consumer observations. Environmental water was taken from the surface layer (ca. 0.5-m depth) and immediately filtered into airtight vials for later measurement of δ^2^H. Subtracting the known discrimination by algae against ^2^H relative to ^1^H [mean ± SD = −161.8 ± 23.0% across published studies (*28*, *61*); measured separately in Virginia lakes as −195.6 ± 34.6% (*11*)] from measurements of environmental water theoretically yielded δ^2^H in phytoplankton. Where δ^13^C and δ^15^N values were desired, we also passed environmental water through cellulose filters with a nominal pore size of 0.8 μm to concentrate POM, which was back-rinsed into vials and dried and ground for isotope analysis. We then estimated the proportion of POM derived from terrestrial and pelagic resources with a two end-member mixing model. Terrestrial δ^2^H was measured, whereas pelagic δ^2^H was estimated from the known discrimination of phytoplankton for different isotopes in environmental water given δ^2^H measured in water. The proportion of POM derived from each of the two resources and δ^13^C and δ^15^N measurements in terrestrial resources allowed us to algebraically solve a mixing model for δ^13^C and δ^15^N in phytoplankton (*6*, *54*). In nine lakes, epiphytic algae were measured as autochthonous resources because they had indistinguishable δ^2^H signatures from phytoplankton (*12*). All organic samples were dried and ground into a homogenous powder for isotopic analysis.

We also collected epilimnetic water samples across the sampling season at weekly to monthly intervals for 73% (*n* = 409) of the consumer observations. These were analyzed for chlorophyll *a* using fluorometry; water color (light absorbance at 440 nm) using spectrophotometry; and DOC, total nitrogen, and total phosphorus using colorimetry. Values were averaged across the sampling period to be representative of overall conditions during the period of consumer growth. Full methods are described elsewhere (*6*–*9*, *11*, *16*, *21*, *23*, *28*, *62*).

### Isotope analysis

Stable isotope ratios of organic samples were measured on isotope-ratio mass spectrometers. Water samples were analyzed for δ^2^H on a cavity ring-down laser spectrometer (*55*).

### Geospatial analysis

We delineated catchment boundaries for each lake by mapping flow direction and accumulation from digital elevation models. Then, by processing digital land use and cover data sets and satellite imagery through the total area that drained into a focal lake, we extracted catchment characteristics for each lake in a given sampling year. The characteristics included area of woody vegetation cover, mean vegetation density, mean soil carbon concentration (0 to 15 cm deep), lake area, lake perimeter, and soil wetness. Generally, catchment delineations and terrain analyses were at a resolution of 30 m, whereas landscape characterization and soil carbon estimates were at resolutions of 250 and 1000 m, respectively. This uniform approach, while at a relatively coarse spatial scale, ensured consistency in both resolution and data sources across lake districts. Our approach also produced very similar results to those derived from higher-resolution catchment delineations provided by individual investigators and an alternative delineation that removed land intersecting other lakes upstream in the same catchment (full details in methods S1).

### Statistical analysis

#### Hypothesis testing with an isotopic mixing model

We tested our five hypotheses by estimating terrestrial resource use (φ_T_) within a Bayesian isotopic mixing model as a direct function of lake water chemistry, catchment characteristics, and consumer identity. Either a one-isotope (δ^2^H only; *n* = 165), two-isotope (δ^13^C-δ^15^N; *n* = 120), or three-isotope (δ^13^C-δ^15^N-δ^2^H; *n* = 274) model was fitted depending on the number of isotopes measured for each consumer observation (total *n* = 559 separate observations). Briefly, the mixing model estimated the relative proportion of terrestrial and aquatic primary production used by each consumer type from stable isotope data and published physiological parameters that varied among consumers. By having only two potential resources, we could fit the same model to all observations, irrespective of the number of isotopes measured; for a one-isotope model, only two resources can be modeled because this reduces to only one unknown variable. The absence of other resources did not bias estimation (methods S3). In addition, for each consumer, the model estimated a unique trophic position, trophic-level fractionation of N, and the contribution of dietary water to δ^2^H ratios from prior information (methods S2) (*7*, *28*, *42*).

Our mixing model had the added benefit of sampling each estimate of terrestrial resource use from a distribution described by a hypothesized network of causal drivers representing our five focal hypotheses. In this network, we predicted allochthony from the availability of both allochthonous and autochthonous resources for the 409 observations with corresponding water chemistry values, allowing an increase in one resource to reduce the effect of the other (that is, an interaction term). The availability of allochthonous resources was equal to the sum of DOC and POC that were terrestrially derived. We had in-lake measurements of DOC that we multiplied against a model-estimated terrestrial proportion but lacked these in-lake observations for POC. Therefore, we described the total terrestrially derived POC as an estimated mean value across lakes that varied with observed catchment and within-lake variables. Using additional techniques to model latent variables, we further informed estimation of allochthonous resources, and hence terrestrial POC, by setting their values to be proportional to observed lake water color. Water color was reported as absorbance at 440 nm, which is a strong indicator of terrestrially derived humic substances (*63*). For autochthonous resources, their availability was equal to measured chlorophyll *a* concentrations. For the remaining 150 observations where no water chemistry was measured, we were still able to estimate terrestrial resource support as a function of consumer preference and season (methods S2). An additional benefit of our mixing model was that we could also incorporate uncertainty in source isotope data and dietary enrichment of δ^2^H and trophic fractionation of δ^15^N into estimates of resource use. Full details of the model are given in methods S2, with reproducible R code in data file S2. Key abbreviations and symbols are listed in table S2.

The model was fitted using Hamiltonian Monte Carlo sampling by calling RStan v2.8 from R v3.2, and we tested for convergence and model misspecification using standard approaches (methods S2). To infer effects, we calculated posterior means and 95% CIs for each parameter by drawing a subset of 1000 simulations. We did not reject hypotheses if 95% CIs for their associated effects excluded 0. All estimated coefficients were standardized to a common scale with a mean of 0 and an SD of 1, so that we could compare the relative importance of different hypotheses.

#### Model validation

The critical test of our mixing model is not only how well it fits our observations but also whether it can unbiasedly recover known parameters of simulated data, specifically consumer isotope ratios, φ_T_, and the effect of lake and catchment-level characteristics on φ_T_. We tested this in different scenarios by randomly sampling δ^13^C, δ^15^N, and δ^2^H values for all 559 consumer observations in our empirical data set from means and variances defining our mixing model (methods S2). First, we tested whether our ability to recover known values when each of the seven potential sources of variation in the mixing model separately varied, as well as the prior means of dietary parameters. Second, we tested whether the model was robust to missing end members. Other resources, such as MOB, certainly contributed to secondary production (*16*, *26*, *28*, *62*, *64*). Therefore, we simulated data with 10, 20, and 40% use of MOB, which had distinct isotopic signatures from terrestrial or pelagic resources (methods S3). A major strength of our approach was that it preserved structure in our original data, while exploring how different sources of variance affected model performance.

## Supplementary Material

http://advances.sciencemag.org/cgi/content/full/3/3/e1601765/DC1

## SUPPLEMENTARY MATERIALS

Supplementary material for this article is available at http://advances.sciencemag.org/cgi/content/full/3/3/e1601765/DC1 method S1. Additional details for geospatial analyses. method S2. Additional details for statistical analysis. method S3. Validation and sensitivity of the Bayesian mixing model. fig. S1. End members used in mixing model and corresponding with each of the 559 consumer observations. fig. S2. Sensitivity of Bayesian mixing model to changes in 7 SDs. fig. S3. Sensitivity of Bayesian mixing model to misinformed dietary priors. fig. S4. Model recovers known parameters despite not accounting for data sets with consumer use of MOB. fig. S5. Predicted isotope ratios versus observed isotope ratios for 559 consumer observations. fig. S6. Prior (light gray curves) and posterior (dark gray curves) of ϕ_T_ for each of the 559 observations organized by consumer type. fig. S7. Lake area distributions globally (black lines) and within our data set (blue lines). fig. S8. DOC distributions from 7514 worldwide lakes. fig. S9. Chlorophyll *a* distribution from 80,012 worldwide lakes. fig. S10. Model recovers known parameters across 100 simulated data sets that span the range of ϕ_T_ (that is, 0 to 1). fig. S11. Catchment area estimated for 147 lakes in our isotope data set. fig. S12. Proportion of each catchment covered with one of four woody vegetation types. fig. S13. Vegetation, geomorphology, and soil characteristics. fig. S14. Catchment area for 46 lakes. fig. S15. Percent overlap in catchments of each of the 46 lakes delineated with three different approaches. fig. S16. Model recovers known parameters despite random noise around the mean effects of covariates predicting the availability of allochthonous resources ξ_*kl*_. fig. S17. Alternate ways of modeling t-OM deposition. table S1. Mean and 95% CIs for model parameter estimates associated with eqs. S1 to S11. table S2. Key symbols and abbreviations used in the text and the Supplementary Materials and Methods. table S3. Reclassification of 2005 North America Land Cover. table S4. Reclassification of 2006 European Land Cover. table S5. Consumer-specific dietary parameters. data file S1. Site-level summary of water quality and catchment characteristics for 147 lakes. data file S2. R code for stable isotope mixing model. References (*65*–*94*)
