# Supplementary material for: Terrestrial support of lake food webs: Synthesis reveals controls over cross-ecosystem resource use
Source: Sci Adv. 2017 Mar 22;3(3):e1601765. doi: 10.1126/sciadv.1601765 (PMC5362171; doi:10.1126/sciadv.1601765)
Supplement: http://advances.sciencemag.org/cgi/content/full/3/3/e1601765/DC1 [file supp_3_3_e1601765__index.html]

Science Advances | Science Advances

## Supplementary Materials

**This PDF file includes:**

- method S1. Additional details for geospatial analyses.
- method S2. Additional details for statistical analysis.
- method S3. Validation and sensitivity of the Bayesian mixing model.
- fig. S1. End members used in mixing model and corresponding with each of the 559 consumer observations.
- fig. S2. Sensitivity of Bayesian mixing model to changes in 7 SDs.
- fig. S3. Sensitivity of Bayesian mixing model to misinformed dietary priors.
- fig. S4. Model recovers known parameters despite not accounting for data sets with consumer use of MOB.
- fig. S5. Predicted isotope ratios versus observed isotope ratios for 559 consumer observations.
- fig. S6. Prior (light gray curves) and posterior (dark gray curves) of ϕT for each of the 559 observations organized by consumer type.
- fig. S7. Lake area distributions globally (black lines) and within our data set (blue lines).
- fig. S8. DOC distributions from 7514 worldwide lakes.
- fig. S9. Chlorophyll a distribution from 80,012 worldwide lakes.
- fig. S10. Model recovers known parameters across 100 simulated data sets that span the range of ϕT (that is, 0 to 1).
- fig. S11. Catchment area estimated for 147 lakes in our isotope data set.
- fig. S12. Proportion of each catchment covered with one of four woody vegetation types.
- fig. S13. Vegetation, geomorphology, and soil characteristics.
- fig. S14. Catchment area for 46 lakes.
- fig. S15. Percent overlap in catchments of each of the 46 lakes delineated with three different approaches.
- fig. S16. Model recovers known parameters despite random noise around the mean effects of covariates predicting the availability of allochthonous resources ξkl.
- fig. S17. Alternate ways of modeling t-OM deposition.
- table S1. Mean and 95% CIs for model parameter estimates associated with eqs. S1 to S11.
- table S2. Key symbols and abbreviations used in the text and the Supplementary Materials and Methods.
- table S3. Reclassification of 2005 North America Land Cover.
- table S4. Reclassification of 2006 European Land Cover.
- table S5. Consumer-specific dietary parameters.
- References (*65–94*)

Download PDF

**Other Supplementary Material for this manuscript includes the following:**

- data file S1 (.csv). Site-level summary of water quality and catchment characteristics for 147 lakes.
- data file S2 (.txt). R code for stable isotope mixing model.

**Files in this Data Supplement:**

- Adobe PDF - 1601765\_SM.pdf
