# Supplementary material for: Terrestrial support of lake food webs: Synthesis reveals controls over cross-ecosystem resource use
Source: Sci Adv. 2017 Mar 22;3(3):e1601765. doi: 10.1126/sciadv.1601765 (PMC5362171; doi:10.1126/sciadv.1601765)
Supplement: http://advances.sciencemag.org/cgi/content/full/3/3/e1601765/DC1 [file 1601765_SM.pdf]

Science Advances
AAAS

advances.sciencemag.org/cgi/content/full/3/3/e1601765/DC1

Supplementary Materials for

Terrestrial support of lake food webs: Synthesis reveals controls over cross-ecosystem resource use

Andrew J. Tanentzap, Brian W. Kielstra, Grace M. Wilkinson, Martin Berggren, Nicola Craig, Paul A. del Giorgio, Jonathan Grey, John M. Gunn, Stuart E. Jones, Jan Karlsson, Christopher T. Solomon, Michael L. Pace

Published 22 March 2017, *Sci. Adv.* **3**, e1601765 (2017)
DOI: 10.1126/sciadv.1601765

The PDF file includes:

- method S1. Additional details for geospatial analyses.
- method S2. Additional details for statistical analysis.
- method S3. Validation and sensitivity of the Bayesian mixing model.
- fig. S1. End members used in mixing model and corresponding with each of the 559 consumer observations.
- fig. S2. Sensitivity of Bayesian mixing model to changes in 7 SDs.
- fig. S3. Sensitivity of Bayesian mixing model to misinformed dietary priors.
- fig. S4. Model recovers known parameters despite not accounting for data sets with consumer use of MOB.
- fig. S5. Predicted isotope ratios versus observed isotope ratios for 559 consumer observations.
- fig. S6. Prior (light gray curves) and posterior (dark gray curves) of φ_T for each of the 559 observations organized by consumer type.
- fig. S7. Lake area distributions globally (black lines) and within our data set (blue lines).
- fig. S8. DOC distributions from 7514 worldwide lakes.
- fig. S9. Chlorophyll *a* distribution from 80,012 worldwide lakes.
- fig. S10. Model recovers known parameters across 100 simulated data sets that span the range of φ_T (that is, 0 to 1).
- fig. S11. Catchment area estimated for 147 lakes in our isotope data set.
- fig. S12. Proportion of each catchment covered with one of four woody vegetation types.
- fig. S13. Vegetation, geomorphology, and soil characteristics.

- fig. S14. Catchment area for 46 lakes.
- fig. S15. Percent overlap in catchments of each of the 46 lakes delineated with three different approaches.
- fig. S16. Model recovers known parameters despite random noise around the mean effects of covariates predicting the availability of allochthonous resources ξ_{kl}.
- fig. S17. Alternate ways of modeling t-OM deposition.
- table S1. Mean and 95% CIs for model parameter estimates associated with eqs. S1 to S11.
- table S2. Key symbols and abbreviations used in the text and the Supplementary Materials and Methods.
- table S3. Reclassification of 2005 North America Land Cover.
- table S4. Reclassification of 2006 European Land Cover.
- table S5. Consumer-specific dietary parameters.
- References (*65–94*)

Other Supplementary Material for this manuscript includes the following:
(available at advances.sciencemag.org/cgi/content/full/3/3/e1601765/DC1)

- data file S1 (.csv). Site-level summary of water quality and catchment characteristics for 147 lakes.
- data file S2. (.txt). R code for stable isotope mixing model.

method S1. Additional details for geospatial analyses.

Data sources

Catchment and lake delineation

We obtained digital elevation models (DEMs) from two sources that allowed us to delineate catchments. For each lake, DEMs were obtained as 1 arc-second × 1 arc-second pixel resolution (approximately 30 m) tiles from NASA's Shuttle Radar Topography Mission (SRTM) (*65, 66*). These SRTM tiles were retrieved from USGS EarthExplorer, courtesy of the NASA EOSDIS Land Processes Distributed Active Archive Center, USGS/Earth Resources Observation and Science Center, Sioux Falls, South Dakota, earthexplorer.usgs.com. For the 9 Swedish lakes, no SRTM tiles were available so we obtained tiles from EU-DEM, a 1 arc-second × 1 arc-second fusion of SRTM and the Advanced Spaceborne Thermal Emission and Reflection Radiometer (ASTER) Global DEM, produced using Copernicus data (*67*).

Several data sources were used for obtaining lake boundaries. Most boundaries (n = 103) were obtained by selecting polygons from OpenStreetMap data in QGIS 2.8.2 (*68*). Due to poor quality OpenStreetMap data for lakes within Sudbury and Dorset, Ontario (n = 11), we extracted those boundaries from The North American Atlas Lakes and Rivers (*69*). Similarly, we used lake boundaries delineated courtesy of the Malcolm Knapp Research Forest for British Columbia, Canada lakes (n = 7). An additional lack of OpenStreetMap data for 26 lakes, led us to digitize lake boundaries manually using the OpenLayers plugin implementation of Google Maps/Bing Aerial satellite imagery in QGIS.

Catchment characteristics

We obtained estimates of land cover from two sources both at a 250 m pixel resolution. For North America, we obtained the ‘2005 North American Land Cover at 250 m spatial resolution’ product of the North American Land Change Monitoring System (*70*). For Europe, we obtained the Corine Land Cover 2006 image version 17 (*71*). Land cover classification schemes differed slightly so we aggregated categories to the lowest taxonomy possible (tables S3 and S4).

We obtained estimates of vegetation density at a 250 m resolution by extracting the Normalized Difference Vegetation Index (NDVI) from Moderate-Resolution Imaging Spectroradiometer MOD13Q1 tiles (*72*). All tiles were accessed for a given sampling year, generally two tiles per month, when possible (dataset date ranges: 2001 – 2014). Tiles were retrieved using the packages *MODIS* v0.10-23/r490 and *rgdal* v0.9-2 in *R* v3.1.

We averaged estimates of soil organic carbon (fine earth fraction <2mm) at 0-5 and 5-15 cm depths for 1,000 m resolution pixels generated by the ISRIC – World Soil ‘SoilGrids1km’ database (*73*, *74*). The reported cross-validation explanatory power of the predictions was 29% (*73*).

Finally, we extracted climatic data at a 1,000 m resolution from the WorldClim database (*75*). Values are averages between 1950 and 2000 based on spatial interpolation amongst 24,542 weather stations (for mean temperatures).

Catchment delineation

We defined a catchment as the area that drained into a focal lake. This first required estimating flow direction using the D8 algorithm and accumulation grids on each DEM from the SRTM or EU-DEM using the 'Breached Depressions' algorithm in WhiteBox Geospatial Analysis Tools v3.2.2 (*76*). We then used the ‘Watershed’ algorithm to determine pixels that contributed to each lake based on the flow direction grid. The entire lake shape was treated as a ‘pour point’ by converting polygons to gridded representations fit to the DEMs. A pour point is a pixel, or in this case, a set of pixels into which all pixels accumulate based on the flow direction algorithm. By treating an entire lake as the pour point, we allowed for multiple inlets. This also accounted for the fact that SRTM 1 arc-second data comes pre-conditioned such that vertical height is flattened across the extent of waterbodies. Sometimes, this meant that the extent of a waterbody in SRTM 1 arc-second data was over- or under-estimated compared to our lake boundaries causing drainage to be localized and unrepresentative of known catchment boundaries. We found that applying a buffer of 0.00025° (ca. 28 m at equator) to each lake was necessary to reduce this effect.

Landscape characterization

We summarized lake and catchment areas using an equal area world projection that ensured these were comparable across the entire dataset (Eckert IV, EPSG: 54012).

For all landscape characterizations, we separated grids and polygons into North America and European datasets, and re-projected them to North America Albers Equal Area Conic (EPSG: 102008) and Europe Albers Equal Area Conic (EPSG: 102008) projections. For a given lake, we then summarized land cover as proportions of different categories within the catchment (tables S3 and S4). We summarized vegetation density as the weighted mean of NDVI values within the catchment from a maximum composite of all tiles available for a given year (i.e., for a given pixel, the maximum value across all tiles was retained). The weighted mean allowed an individual pixel’s contribution to the mean value to be proportional to the area it occupied within a focal catchment, thereby minimizing the effects of pixel size on summary statistics at polygon edges. We similarly derived the weighted mean of soil C within catchments. Using slopes and flow accumulation from each DEM, we also calculated the

Compound Topographic Index in Whitebox (CTI = ln[accumulation / *tan* slope]). CTI estimates the tendency for a pixel to accumulate water, i.e. soil wetness (*77*), and we summarized values across pixels with a weighted mean within the catchment.

Do alternative methods for catchment delineation influence geospatial characteristics?

We tested whether differences in methodology and DEM resolution influenced our catchment delineations and their extracted characteristics. We first compared our catchment delineation (hereafter 'aggregate catchments') with one where we placed focal lakes in the context of other lakes within the region (hereafter 'context catchments'). To derive context catchments, we obtained waterbodies $\geq$ 496 m^2 (the smallest waterbody in the dataset) from the OpenStreetMap multi-polygon data described above and delineated their catchments. All land contributing to a given lake that did not first meet a waterbody $\geq$ 496 m^2 was subsequently included in the catchment, resulting in smaller catchments than estimated from the aggregate approach. By contrast, the aggregate catchments included all land contributing to a given lake based on the D8 algorithm in WhiteBox. Based on flow accumulation, a given lake's catchment could therefore be the accumulation of several catchments upstream.

We also considered catchments delineated by the different research groups that contributed stable isotope data (hereafter 'investigator catchments'). These were delineated at various resolutions (2-10 m). In total, catchments were compiled for 46 sites across British Columbia, Michigan, Sweden and Wisconsin, and we characterized their landscapes using aforementioned methods.

The different approaches for delineating catchments were all relatively consistent with each other. First, we found that both the aggregate (AG) and context (CO) catchments produced similar geospatial characteristics, except for in large flat catchments common in Sweden and Wisconsin. In these regions, focal catchments were often connected upstream to other lakes $\geq$ 496 m^2 in size, leading to much larger estimates of catchment area using the aggregate than context approach (fig. S11). Vegetation was also very similar between the two approaches to catchment delineation (figs. S12, S13).

Second, we found that catchment area derived from individual investigators, often at higher spatial resolution, corresponded well with both of our aggregate (AG) and context (CO) delineations (fig. S14). Both the AG and CO catchments were similarly correlated with the investigator values (Pearson's correlation r = 0.76 and r = 0.80, respectively; 95% confidence interval for difference between correlation coefficients overlapped zero). However, the AG and investigator (IN) catchments overlapped by <1 to 15% more (95% confidence intervals) than the IN did with the CO (paired *t*-test comparing mean proportion of overlapping polygons between AG and IN catchments with CO and IN: t_{45} = -2.07, p = 0.044). In general, the AG-IN catchments overlapped across 55% ± 30% (mean ± SD) of pixels, as compared

with the 47% ± 23% overlap of the CO-IN catchments (fig. S15).

method S2. Additional details for statistical analysis.

Isotopic mixing model

We let the ratios of $\delta^{13}C$, $\delta^{15}N$, and $\delta^{2}H$ in observation *i* of consumer *j* from site *k* measured by research group *g* in season *s* be described by a linear mixture of terrestrial $\mathbf{t}_{gks}$ and pelagic $\mathbf{p}_{gks}$ resources, each being a vector of length *m* with elements respectively corresponding to mean values for each of the *l* = 3 isotopes that were measured. We could then sample isotopic ratios from a multivariate normal distribution with a mean vector $\boldsymbol{\mu}_{gijks}$ and $l \times l$ matrix $\boldsymbol{\Sigma}_{gijks}$ to estimate the relative contributions of terrestrial ($\phi_{\mathrm{T}gijks}$) and pelagic ($\phi_{\mathrm{P}gijks}$) resources to each consumer observation

$$\boldsymbol{\mu}_{gijks} = \phi_{\mathrm{T}gijks}\mathbf{t}_{gks} + \phi_{\mathrm{P}gijks}\mathbf{p}_{gks} \qquad \text{(S1)}$$

$$\phi_{\mathrm{T}gijks} + \phi_{\mathrm{P}gijks} = 1$$

We propagated the uncertainty associated with $\mathbf{t}_{gks}$ and $\mathbf{p}_{gks}$ into $\boldsymbol{\Sigma}_{gijks}$ by summing the product of each of $\phi_{\mathrm{T}gijks}$ and $\phi_{\mathrm{P}gijks}$ and their respective observed variances, and adding this to an estimated *l* length vector of residual errors $\boldsymbol{\varepsilon}$, wherein each element was sampled from a uniform distribution between 0 and 20. This generated a vector of standard deviations $\boldsymbol{\sigma}_{gijks}$ that we multiplied with an estimated $l \times l$ correlation matrix $\boldsymbol{\Omega}$ to derive $\boldsymbol{\Sigma}_{gijks}$.

For estimating $\delta^{15}N$ ratios, we modified eqn S1 to account for progressive enrichment of $\delta^{15}N$ as trophic levels increase (*79*)

$$\boldsymbol{\mu}_{2[gijks]} = \phi_{\mathrm{T}gijks}\mathbf{t}_{2[gks]} + \phi_{\mathrm{P}gijks}\mathbf{p}_{2[gks]} + \Delta_j \qquad \text{(S2)}$$

This first required estimating the number of trophic levels that each consumer *j* was above primary producers from a normal distribution with a consumer-specific mean and universal SD of 0.1 (table S5). We then estimated trophic enrichment Δ_j in consumer *j* from a normal distribution with mean equal to the product of τ_j and the mean per-trophic-level isotopic enrichment of N, which was 2.52 (SD = 1.46) based on data for 40 taxa (*80*). The SD for the prior distribution of Δ_j propagated error in both of its coefficients and was equal to $[(2.52 \times 0.1) + (\tau_j \times 1.46)]^{1/2}$.

We also modified eqn S1 to account for the fact that each consumer *j* obtains a proportion ω_j of $\delta^{2}H$ from water consumed in their diet (*55*), which has a $\delta^{2}H$ ratio of w_{gks}

$$\boldsymbol{\mu}_{3[gijks]} = \omega_j w_{gks} + (1 - \omega_j)(\phi_{\mathrm{T}gijks}\mathbf{t}_{3[gks]} + \phi_{\mathrm{P}gijks}\mathbf{p}_{3[gks]}) \quad \text{(S3)}$$

ω_j was estimated from a Beta distribution to constrain values between 0 and 1 and had a mean equal to $1 - (1 - \omega_{\mathrm{P}j})^{\tau_j}$ to allow for trophic compounding of dietary water (*79*). $\omega_{\mathrm{P}j}$ was the per-trophic-level contribution of environmental water to consumer *j* and was derived from relatively controlled studies of consumer diets (table S5). We incorporated uncertainty in both $\omega_{\mathrm{P}j}$ and τ_j into the prior variance of ω_j using generalised equations for propagating normally-distributed errors (*81*).

Structural equation model of allochthonous support

We tested our five hypothesised drivers of allochthonous support by estimating a series of equations S4-S11 that described a network of presumed cause-and-effect associations (*82*, *83*). The statistical basis of this network involved sampling $\phi_{\mathrm{T}gijks}$ from a Beta distribution to constrain values between 0 and 1 and simplify to only one unknown variable in eqn S1 in instances where only one isotope (δ^2H) was observed; as $\phi_{\mathrm{P}gijks} = 1 - \phi_{\mathrm{T}gijks}$. The Beta distribution was given a mean λ_{gjks} and estimated SD σ_{T} that was then related to our study hypotheses.

(i) Consumer preference hypothesis

We tested whether consumers differed in their estimated mean levels of allochthonous resource use $\alpha_{1[j]}$ and/or estimated response $\beta_{1[j]}$ to an increasing availability of allochthonous resources ξ_{gk} by allowing terrestrial resource use λ_{gjks} to vary with each consumer *j*

$$\mathrm{logit}(\lambda_{gjks}) = \alpha_{1[j]} + \beta_{1[j]}\xi_{gk} + \beta_{2[j]}\mathrm{CHLA}_{gk} + \beta_{3[j]}\xi_{gk}\mathrm{CHLA}_{gk} + \eta_g + \alpha_{2[s]} \quad \text{(S4)}$$

accounting for the fact that λ_{gjks} also depends on the amount of autochthonous resources CHLA_{gk} in each site *k* across the time period observed by research group *g* and measured as lake water chlorophyll *a* concentration, and the contribution of allochthonous resources towards terrestrial resource use may attenuate as more favourable autochthonous resources become available (i.e. $\xi_{gk} \times \mathrm{CHLA}_{gk}$ interaction). We also accounted for variation in λ_{gjks} outside of the summer season by allowing the intercept to vary with strength $\alpha_{2[s]}$ and simply due to the slightly different field methods of each research group by randomly sampling a value of η_g from a zero-centered normal distribution with estimated SD, analogous to treating research group as a ‘blocking factor’. The logit constraint in eqn S4 ensured that the mean proportional use of terrestrial resources laid between 0 and 1. Any two consumers therefore differed in their terrestrial resource use if the absolute 95% credible interval (CI) for their difference in either α_1 or β_1 values was >0.

For 150 of the 559 consumer observations that lacked associated water quality data, we estimated a term υ_{gk} that accounted for the variation around consumer means associated with the summed effect of allochthonous and autochthonous resources

$$\mathrm{logit}(\lambda_{gjks}) = \alpha_{1[j]} + \upsilon_{gk} + \eta_g + \alpha_{2[s]} \quad \text{(S5)}$$

As all our covariates were centered to a mean of zero (see *Model estimation* section), we could impute missing values of $\beta_{1[j]}\xi_{gk} + \beta_{2[j]}\mathrm{CHLA}_{gk} + \beta_{3[j]}\xi_{gk}\mathrm{CHLA}_{gk}$ by sampling υ_{gk} from a normal distribution with estimated mean and SD. This did however mean we could not use these 150 observations to test our other hypotheses, though we could estimate $\phi_{\mathrm{T}gijk}$.

(ii) Seasonality hypothesis

We tested whether terrestrial resource use increased outside of summer, which contained most of our observations (n = 429), by estimating $\alpha_{2[s]}$ in eqns S4-S5 for each non-summer season. We could not reject our hypothesis if 95% CIs were positive and excluded zero.

(iii) Catchment deposition hypothesis

We tested whether the use of allochthonous resources averaged across all consumers increased with the availability of those resources, and whether those resources themselves increased with vegetation and soil carbon and/or the delivery potential of the surrounding catchment. In the first instance, each value of $\beta_{1[j]}$ in eqn S4 was estimated from a normal distribution with mean B and estimated SD σ_B. A 95% CI for B that was positive and excluded zero suggested $\phi_{\mathrm{T}gijks}$ increased with ξ_{gk} on average across all consumers.

To test the second part of the catchment deposition hypothesis, we related ξ_{gk} to vegetation, soil, and geomorphological characteristics through organic carbon inputs. Allochthonous resources in lake water are a sum of the proportions of dissolved (DOC) and particulate (POC) organic carbon that are terrestrially derived and so the expected value of ξ_{gk} was equal to the sum of log-transformed values of these two pools

$$\xi_{gk} = \beta_4\mathrm{DOC}_{gk} + \beta_5\mathrm{POC}_{gk} \quad \text{(S6)}$$

DOC_{gk} was then related to vegetation and soils by modelling it from a lognormal distribution with estimated mean κ_{gk} and SD σ_κ. κ_{gk} was equal to the sum of an estimated mean DOC concentration across lakes α_3, which accounts for a consistent autochthonous contribution towards measured DOC, and the estimated effects of the mean vegetation density in the surrounding catchment NDVI_{gk}, measured by the normalised difference vegetation index, mean catchment soil carbon density at a 0-15 cm depth SOIL_k, and log-transformed wetness

in the surrounding catchment, measured as the mean compound topographic wetness index $WETS_k$. We also accounted for variation among sites in DOC simply due to geomorphology and temperature-dependent terrestrial primary production and microbial decomposition that stimulates DOC release (*51*, *84*)

$$\kappa_{gk} = \alpha_3 + \beta_6 \mathrm{NDVI}_{gk} + \beta_7 \mathrm{SOIL}_k + \beta_8 \mathrm{WETS}_k + \beta_9 \mathrm{LPLA}_k + \beta_{10} \mathrm{TEMP}_k \qquad \text{(S7)}$$

where $LPLA_k$ was the log-transformed ratio of lake perimeter to area, indicating exposure to the surrounding catchment, and $TEMP_k$ was the long-term (1950-2000) mean daily air temperature during the warmest quarter of the year (*75*). Previous global-scale analyses have shown that lake water DOC increases with both soil carbon concentrations and wetland coverage (*51*, *43*), but we used $WETS_k$ in our analysis rather than wetland cover because it can better detect closed-canopy wetlands that are omitted from traditional vegetation classifications (*85*). As for POC_{gk}, it was not directly observed and so we let $\beta_5 POC_{gk}$ – the total terrestrially derived POC – equal the sum of an estimated mean value across lakes α_4 that varied with NDVI, the cube-rooted area of woody vegetation in the surrounding catchment (sum of broadleaf, coniferous, mixed forest, and shrubland vegetation; tables S3 and S4) per metre shoreline $WOOD_k$, $WETS_k$, and $LPLA_k$. We also let $\beta_5 POC_{gk}$ vary with $TEMP_k$ to account for less POC availability where mineralisation of larger particles is promoted by warmer ice-free temperatures (*86*)

$$\beta_5 \mathrm{POC}_{gk} = \alpha_4 + \beta_{11} \mathrm{NDVI}_{gk} + \beta_{12} \mathrm{WOOD}_k + \beta_{13} \mathrm{WETS}_k + \beta_{14} \mathrm{LPLA}_k + \beta_{15} \mathrm{TEMP}_k + \zeta_{gk} \qquad \text{(S8)}$$

α_4 (i.e. the mean value) reduced to zero where all the covariates were scaled to a mean of zero and ζ_{gk} was normally distributed error that was sampled from an estimated SD σ_ξ. Positive 95% CIs that then excluded zero for B as well as β_{11}, β_{12}, β_{13}, β_{14} or β_4 and any of β_6, β_7, β_8, β_9 did not allow us to reject the catchment deposition hypothesis.

As the availability of allochthonous resources was a latent (i.e. unmeasured) variable, due to its inclusion of $\beta_5 POC_{gk}$, we further defined ξ_{gk} on the scale of our observed data by letting it be proportional to lake water colour and total nitrogen (TN). Both water colour and TN were explicitly modelled as correlated variables from a multivariate lognormal distribution with estimated correlation matrix, vector of SD, and vector of means $\boldsymbol{\gamma}_{gk}$ with respectively corresponding elements

$$\boldsymbol{\gamma}_{1[gk]} = \alpha_5 + \xi_{gk} \qquad \text{(S9)}$$

$$\boldsymbol{\gamma}_{2[gk]} = \alpha_{6[k]} + \beta_{16} \xi_{gk} \qquad \text{(S10)}$$

where α_5 was mean water colour across lakes, $\alpha_{6[k]}$ was mean TN that varied depending on the presence of urban land use in the surrounding catchment, and β_{15} was the estimated scaling of TN with ξ_{gk}.

(iv) Favourable resources hypothesis

Allochthonous resource use in lakes can decline as autochthonous resources that have higher nutritional value become more available. We could not reject this hypothesis if the 95% CI for β_2 or β_3 in eqn S4 was negative and excluded zero.

(v) Algal subtraction hypothesis

Terrestrially derived organic matter can fertilize algal growth by supplying limiting nutrients, especially nitrogen, and thus strengthen the favourable resources hypothesis. However, this depends on whether these terrestrial resources shade the water column and dampen algal growth. We tested the strength of these two mechanisms by modelling lake water chlorophyll *a* concentrations from a lognormal distribution with an estimated SD and mean θ_{gk} that varied with lake water colour CL_{gk} and total nitrogen concentrations TN_{gk}. The benefit of TN_{gk} for algal growth will also depend on whether total phosphorus TP_{gk} is non-limiting and may be stronger in such cases (i.e. $TN_{gk} \times TP_{gk}$ interaction). TP_{gk} on its own should also account for variation in algal productivity. We then estimated a mean chlorophyll *a* concentration across lakes α_7 and let this vary with log-transformed values of the covariates

$$\theta_{gk} = \alpha_7 + \beta_{17}CL_{gk} + \beta_{18}TN_{gk} + \beta_{19}TP_{gk} + \beta_{20}TN_{gk}TP_{gk} \qquad \text{(S11)}$$

We did not reject the subsidy hypothesis if the 95% CI for β_{18} was positive and excluded zero, whilst we did not reject the shading hypothesis if the 95% CI for β_{17} was negative and excluded zero.

Model estimation

We simultaneously fitted eqns S1-S11 to our empirical dataset by simulating four Markov Chains of 5,000 iterations with a burn-in of 4,000 runs. All regression coefficients (i.e. α, β, B) and SDs (i.e. σ) were assigned uninformative and weakly informative normal priors on the scale of our observations, either $\sim N(0,10)$ or $\sim N(0,1)$ respectively (*87*). Priors for correlation matrices were sampled from LKJ distributions that placed almost uniform support over the estimated correlations (shape parameter η = 2), with the density slightly more concentrated around the identity matrix (*88*). Model code is given in supplementary data file S2.

Convergence of MCMC chains was assessed by visually inspecting chain traces and calculating the potential scale reduction factor *psrf* for the posterior distribution of each parameter. *psrf* predicts the relative change in a parameter's CIs if models are run infinitely longer. All values were <1.1, which is considered acceptable (*87*). We also ensured that the effective number of simulation draws n_{eff}, a measure of independence amongst the subset of simulations, always exceeded 800 (*87*).

We used a graphical modelling approach to evaluate the SEM (*89*). This was relatively straightforward given that we had only one theoretical construct in our model and so there was no need to ensure that different latent variables measured different processes. There was also only one potentially missing linkage from the latent variable to a modelled observed variable (chlorophyll *a*), but this had no real support (Spearman's rank correlation between residuals for chlorophyll *a* and ξ_{gk}: $\rho = 0.05$; $p = 0.633$). Finally, we graphically inspected associations between observed and predicted values, and between model predictions and residuals, for each modelled variable to ensure consistency between our causal mechanism and measured data. Overall, the graphical modelling approach showed strong data-model consistency, supporting the use of our SEM for inference of causal pathways.

method S3. Validation and sensitivity of the Bayesian mixing model.

Data simulation

We generated new observations of $\varphi_{\mathrm{T}ijkl}$ for each observation *i* of consumer *j* from site *k* at time *l* given known effects of water chemistry and catchment characteristics. This involved parametrising the Beta described in methods S2 with the effective sample size and consumer-specific values of mean $\varphi_{\mathrm{T}ijkl}$ and effects of water chemistry and catchment characteristics estimated in the main text. Given that the availability of allochthonous resources ξ_{kl} was also unmeasured, we generated new values for this parameter by sampling from a normal distribution that assumed a 1:1 scaling with the mean association between water colour and predictors of organic carbon, as described by eqns S6 and S9 in methods S2. Altering this 1:1 assumption in data generation but not the modelling by introducing random noise into the true effects did not influence parameter recovery (fig. S16). Finally, we also simulated new trophic positions (τ), contributions of environmental water to consumer isotope ratios (ω), and trophic enrichment of N (Δ_j) from the distributions described in table S5. Critically, exact values for all the parameters are unimportant as we later vary them and test how this changes the ability of the Bayesian mixing model (BMM) to recover known values.

How much does uncertainty in each variance parameter influence model fit?

We tested how well the BMM recovered 'true' values of φ_T and focal effects with 95% CIs that excluded zero in the main text as each of the following sources of variation changed:

- residual error associated with φ_T (σ_{res})
- residual error in trophic position of each consumer (σ_τ)
- residual error in per-trophic-level contribution of dietary water to δ^2H (σ_ω)
- residual error associated with individual consumer isotope observations (σ_O)
- residual error associated with environmental water samples (σ_W)
- residual error associated with terrestrial end member observations (σ_T)
- residual error associated with pelagic end member observations (σ_P)

For each source, we varied the associated SD along 10 equally-spaced intervals from 0–300% of its original value. We then simulated 10 datasets at each interval by randomly sampling new values for φ_T, ξ_{kl}, Δ_j, τ, and ω and used these to sample new isotope values for each consumer observation in our empirical dataset from the distributions described by eqns S1-S3 in methods S2. The distributions were parameterised with empirically-observed means and variances of environmental water and end members, propagating their associated uncertainty. Across the 10 simulations in each interval, we fitted the BMM with the original means and variances of environmental water and end members and calculated relative bias between predicted and observed values of φ_T and all of the following effects in the structural equation model: allochthonous resources on φ_T, DOC on ξ_{kl}, and NDVI and ratio of lake perimeter to lake area on POC. We did not test how variation in the residual error associated with Δ_j influenced our predictions as this estimate was already reasonably large (58% of mean) and derived from a large dataset for which there was little reason to expect that it was estimated with error (see methods S2).

We found that bias in model predictors and φ_T were relatively invariant to increasing uncertainty in the seven variance parameters (fig. S2). All simulations were within the range of values observed when empirical variances were set at 100% of their original σ's (i.e. overlap between bars and grey polygon). The primary exception was that we found greater variability in bias as residual error in individual consumer observations increased to relatively high levels (>200%; pink lines in figs. S2b,d).

How much does bias in prior means influence model fit?

φ_T may be estimated biasedly if the relatively informative prior distributions that we set for the dietary parameters of trophic position (τ) and per-trophic-level contribution of environmental water to consumer isotope ratios (ω_P) in table S5 differed from the 'true' values of these parameters that generated the empirical data. We therefore tested the sensitivity of φ_T to this potential source of error by varying each of τ and ω_P along 10 equally-spaced intervals from 20–200% of their original values and simulating 10 datasets at each interval as described in the preceding section. Prior SDs remained unchanged (i.e. equal to table S5).

We found that prior expectations for τ or ω_P that considerably deviated from the 'true' values used to generate isotopic ratios of consumers estimated both the effects of covariates on φ_T and φ_T itself as well as when the priors were accurate (i.e. 100% of μ, fig. S3). These results clearly show that misinformed priors for τ or ω_P make little difference in the empirical dataset even where the misinformation is large, and this is unlikely given our data sources.

Do missing end members bias allochthony estimates?

We simulated data as in previous analyses but allowed methane oxidizing bacteria (MOB) to contribute towards isotopic ratios. We considered scenarios in which consumer use of MOB (φ_B) was either 10, 20, or 40%. For the remaining proportion of diet, we retained the mean ratio of terrestrial to phytoplankton resource use observed empirically in the main text (see fig. 2), i.e. $\varphi_T = (1 - \varphi_B) \times 37\%$, allowing for consumer-specific variation (fig. 5 in main text). MOB had mean (SD) values for δ^2H, $\delta^{13}C$, and $\delta^{15}N$ of -200 (15), -60 (10), and 0 (1), respectively (after refs. *16*, *26*, *90*), and we randomly sampled site × time observations from these distributions for each empirical observation. We then tested how φ_T and its associated predictors responded to omitting MOB from the BMM despite MOB contributing to the data generating process.

We found that end members that were unaccounted for in the BMM did not strongly bias estimation of the associations between φ_T and its predictor variables, though φ_T itself was biased when the omitted end members contributed substantially to consumer biomass (fig. S4). The strongest bias in a predictor variable was for the effect of allochthonous resources on φ_T (pink lines in figs. S4b,f,j). However, this was still relatively minor, i.e. median values underestimated by 11-21%. As this bias was negative, it also suggested that the significant effect detected for this parameter in the empirical dataset (i.e. 95% CIs positive and excluding zero in figs. 1b, 3 in the main text) could even be stronger where MOB were utilised as a resource. We also recovered the φ_T used to simulate the data with bias >30% only where $\varphi_B \geq 20\%$ (fig. S4). φ_T was increasingly overestimated as φ_B increased (figs. S4c,g,k), leading to greater bias between predicted and 'true' observations, especially where $\varphi_T < 0.40$ (figs. S4d,h,l). Overall, the fact that simulations with $\varphi_{MOB} = 0\%$ resembled those when $\varphi_{MOB} = 10\%$ much more than when $\varphi_{MOB} = 20$ or 40% (fig. 1 in main text vs fig. S4) suggests that our empirical dataset had a minimal MOB contribution.

Are conclusions robust to alternative indicators of terrestrial organic matter deposition?

We reached similar conclusions to the main text when we considered different ways to estimate terrestrial organic matter (t-OM) deposition. We repeated the analyses described in the main text and methods S2, but replaced the ratio of lake perimeter (LP) to lake area (LA) with the ratio of catchment area (CA) to LA. Larger CA:LA ratios have been positively associated with organic carbon loading in regional studies (*37*, *91-92*), though not across different bioclimatic zones where variation in topography, climate, and hydrology may be

more important (*43, 51*). CA:LA ratios may also have little influence over t-OM export where values are greater than between 6 to 10 (*91*), corresponding with 71 and 57% of our study lakes, respectively. Consistent with this past work, we found that the availability of allochthonous resources influenced φ_T and that we could not reject the catchment deposition hypothesis when terrestrially derived POC considered shoreline influence rather than CA (fig. S17a). We also rejected support for the catchment deposition hypothesis when woody vegetation in surrounding catchments was expressed in absolute area and not per unit shoreline and when NDVI was expressed per CA (figs. S17b-d). We expressly avoided selecting the model reported in the main text from these alternative parameterisations simply on the basis of goodness of fit because we did not want to dredge the data for patterns without strong causal hypotheses. Collectively, our findings suggested that the promotion of allochthony by terrestrial resources depended upon POC and that processes in the immediate vicinity of the shoreline were more important than across the whole of the catchment in enhancing this carbon pool, consistent with past work (*43, 93–94*).

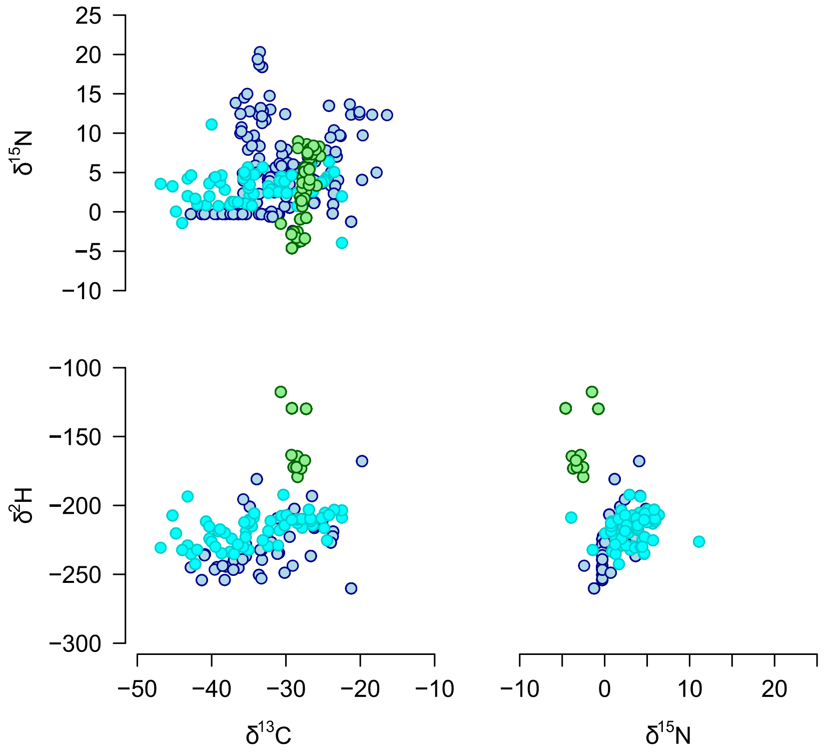

fig. S1. End members used in mixing model and corresponding with each of the 559 consumer observations. Isotopes were measured either for terrestrial resources (green symbols) or pelagic phytoplankton (dark blue symbols), and not all isotopes were measured for each observation. For n = 226 observations of δ^2H, pelagic phytoplankton values were estimated using the discrimination between ^{2}H and ^{1}H during photosynthesis (light blue points). For n = 152 observations of δ^{13}C and δ^{15}N, pelagic phytoplankton values were estimated from isotopic measurements of lake POM (light blue points). This approach first involved finding the proportion of POM derived from terrestrial and pelagic resources given known δ^2H, itself estimated for phytoplankton from the difference between measured environmental water and dietary discrimination of the heavier isotope. δ^{13}C and δ^{15}N of phytoplankton were then solved algebraically in a two-resource mixing model given known proportions and isotope values of POM and the terrestrial resource.

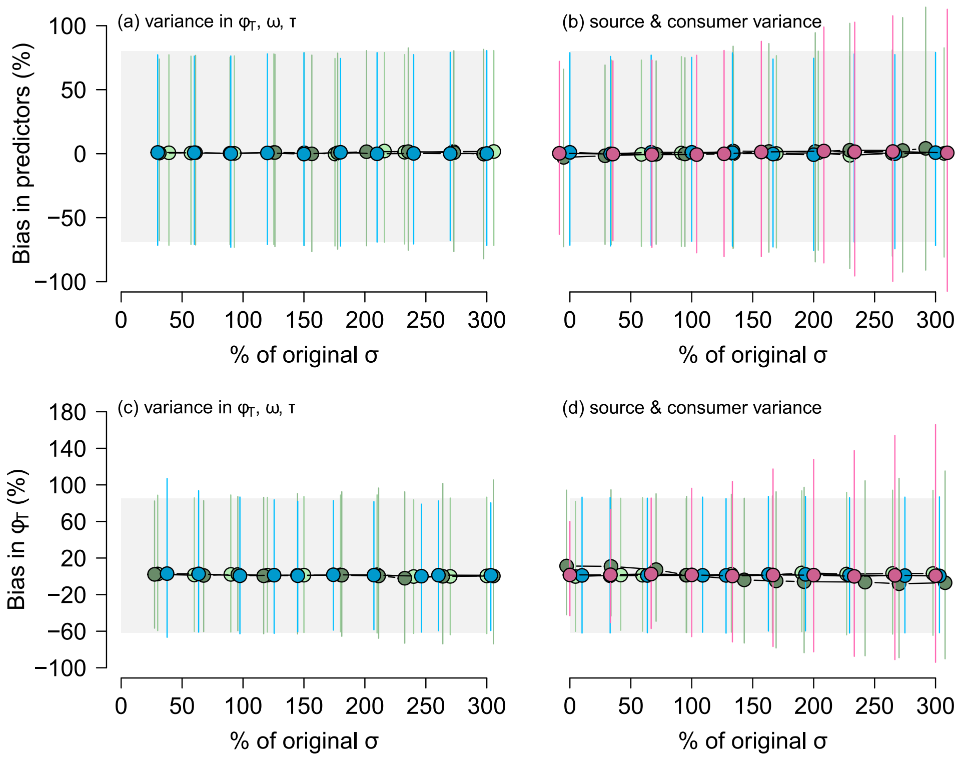

fig. S2. Sensitivity of Bayesian mixing model to changes in 7 SDs. For each of 10 potential changes in a given SD, we fitted the BMM to 10 simulated datasets and calculated the mean percent bias (±95% CI) between observed and predicted (**a**,**b**) effects of covariates in our structural equation model and (**c**,**d**) φ_T. For (a,b), error in each simulation was calculated across all of the following effects of interest: allochthonous resources on φ_T, DOC on ξ_{kl}, and NDVI and ratio of lake perimeter to lake area on POC. We classified parameters as those associated with (a,c) σ_{res} (blue), dietary water σ_ω (dark green) and trophic position σ_τ (light green); and (b,d) source and consumer isotope values: σ_O (pink); σ_W (blue); σ_T (light green); σ_P (dark green). Grey box is 95% CI for 100 simulations where no changes were made to the parameters (100% of original σ from fig. 1 in main text). Zero values could not be used for σ_{res}, σ_ω and σ_τ because the associated Beta distributions would have shape parameters equal to ∞; for σ_O some error was necessary for convergence. Points are jittered along the *x*-axis for clarity.

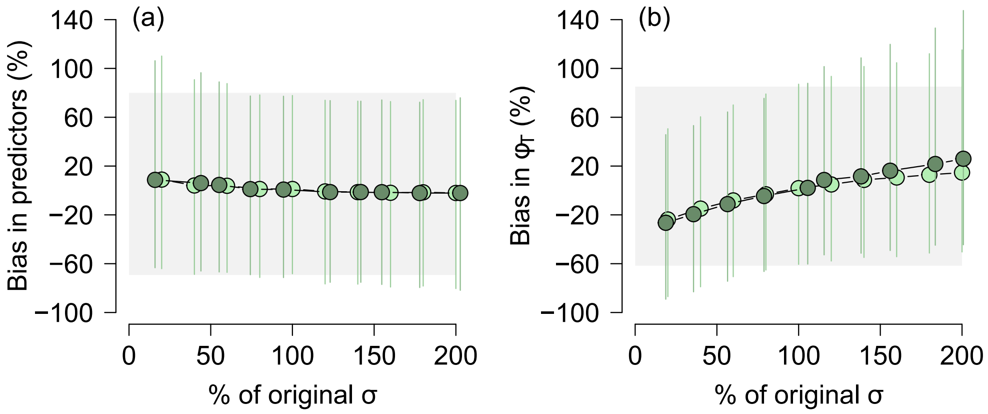

fig. S3. Sensitivity of Bayesian mixing model to misinformed dietary priors. For each of 10 potential changes in the prior mean (μ) of τ (dark green) and ω_P (light green), we fitted the BMM to 10 simulated datasets and calculated the mean percent bias (±95% CI) between observed and predicted (**a**) effect of a catchment characteristic on levels of terrestrial resource use φ_T and (**b**) φ_T. Grey box is 95% CI for 100 simulations where no changes were made (100% of original μ from fig. 5 in main text). Points are jittered along the *x*-axis for clarity.

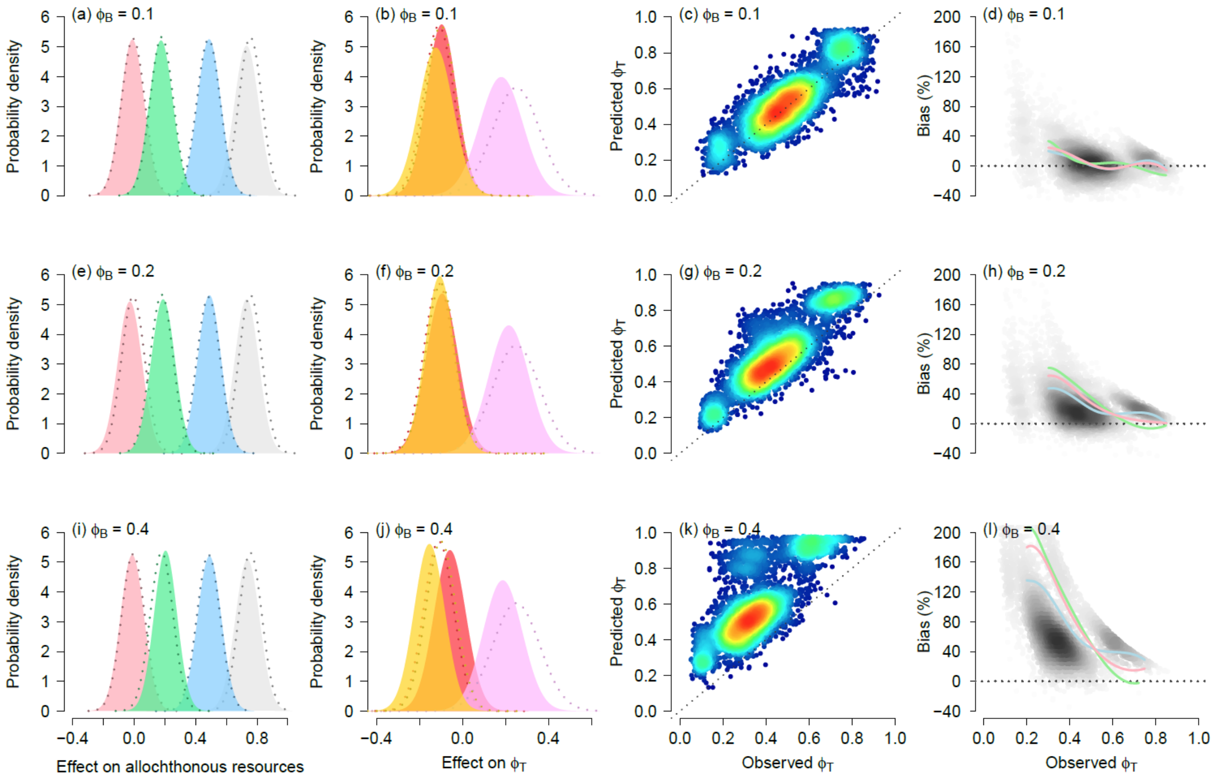

fig. S4. Model recovers known parameters despite not accounting for data sets with consumer use of MOB. Consumer use of MOB was set to **(a-d)** 10%, **(e-h)** 20%, or **(i-l)** 40%. Mean posterior distributions across 10 simulations of the effects of (a, e, i) DOC (gray), NDVI (pink), ratio of lake perimeter to area (blue), and area of woody vegetation per meter shoreline (green) on availability of allochthonous resources; (b, f, j) allochthonous resources (purple), lake chlorophyll *a* (red), and their interaction (orange) on terrestrial resource use (φ_T); dashed lines are known prior distributions. (c, g, k) Mean predicted versus observed (i.e. known) φ_T for 559 consumer observations in each of 10 simulations. Warmer colours indicate greater concentration of points (total n = 5,590). (d, h, l) Percent bias in mean predicted φ_T values. Darker shading indicates greater concentration of points. Lines are splines fitted through observations upon one (δ^2H-only, pink), two (δ^{13}C-δ^{15}N, green), or three (δ^{13}C-δ^{15}N-δ^2H, blue) isotopes.

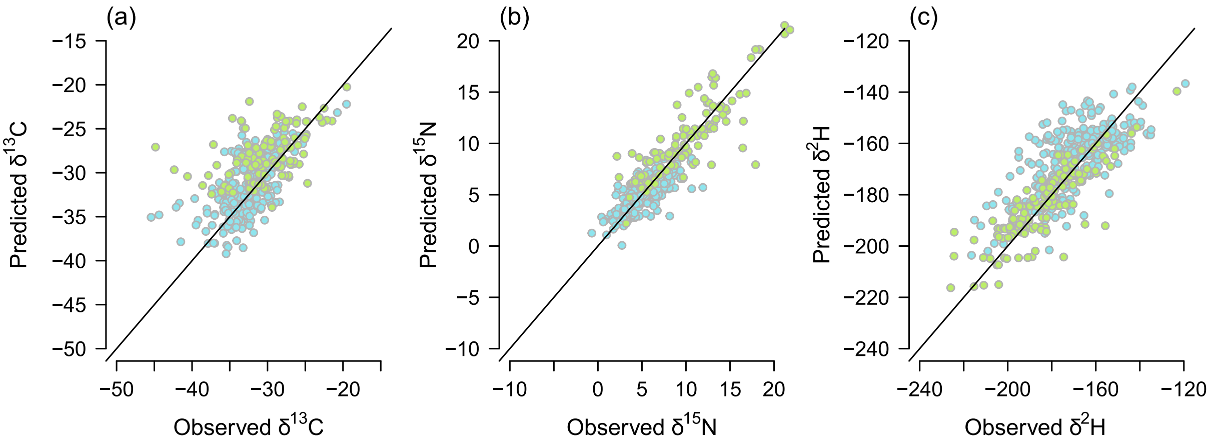

fig. S5. Predicted isotope ratios versus observed isotope ratios for 559 consumer observations. Blue symbols are observations for which (**a**) $\delta^{13}C$, (**b**) $\delta^{15}N$, and (**c**) $\delta^{2}H$ were all measured, while green symbols are those where only (a,b) $\delta^{13}C$ and $\delta^{15}N$ or (c) $\delta^{2}H$ were measured. Bayesian R^2 values for $\delta^{13}C$, $\delta^{15}N$, and $\delta^{2}H$ were 0.23, 0.54, 0.35 respectively in the three-isotope model, 0.18 and 0.71 for $\delta^{13}C$ and $\delta^{15}N$ respectively in the two-isotope model and 0.64 in the $\delta^{2}H$ model. Bayesian R^2 values for all consumer × isotope observations in the three- and two-isotope models were 0.99 and 0.98, respectively.

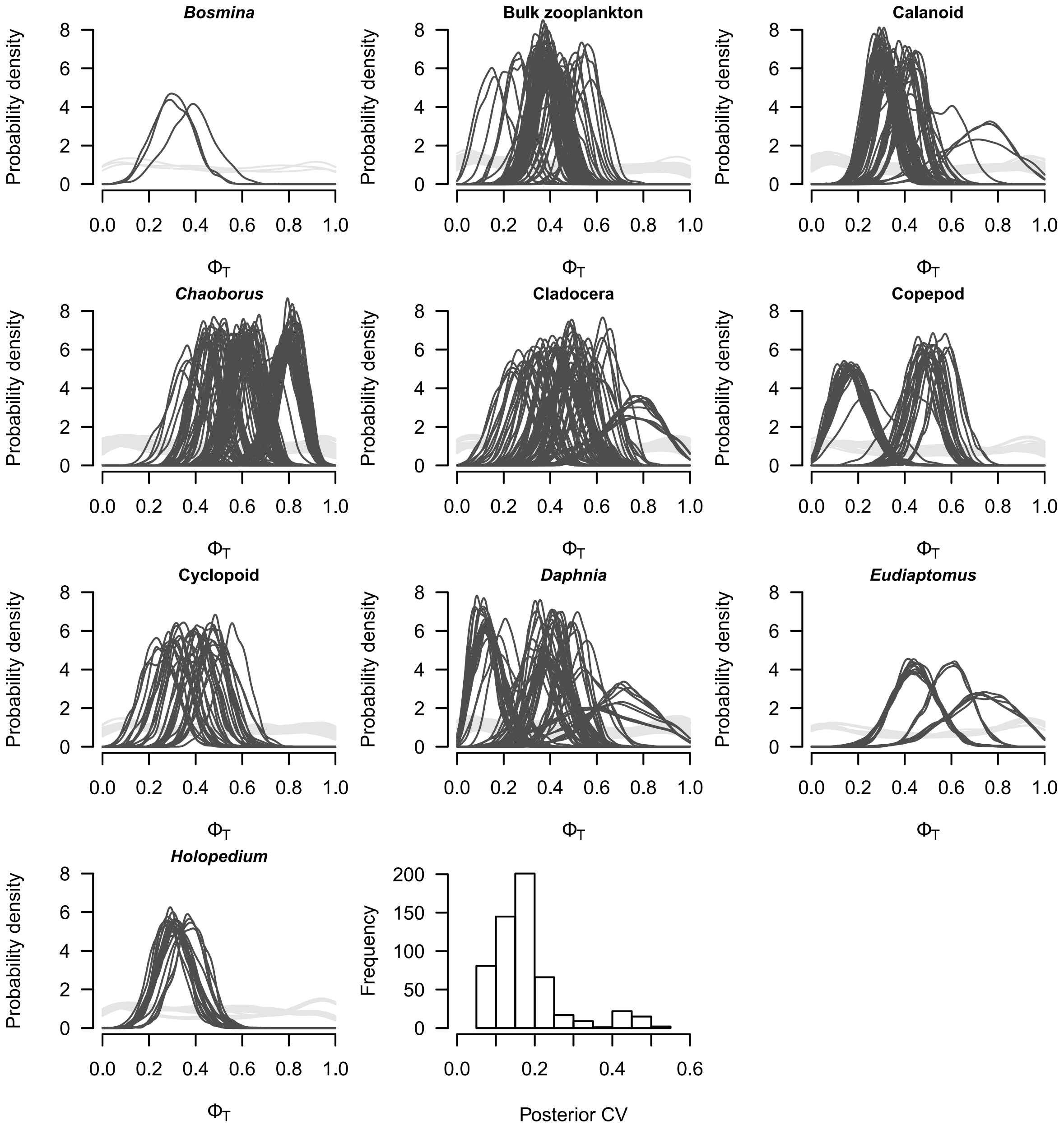

fig. S6. Prior (light gray curves) and posterior (dark gray curves) of φ_T for each of the 559 observations organized by consumer type. Histogram is the coefficient of variation (CV) calculated across all posterior distributions.

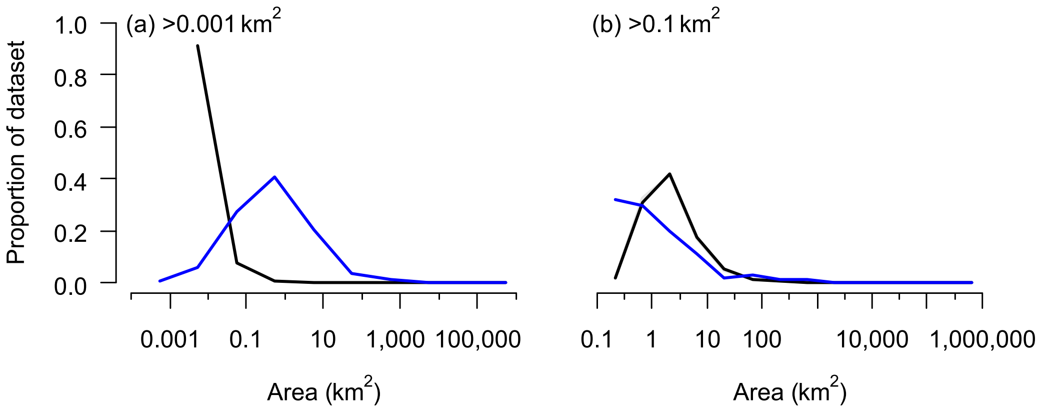

fig. S7. Lake area distributions globally (black lines) and within our data set (blue lines). Global distributions were calculated either (**a**) from eqn 7 in ref. (*50*) for lakes worldwide >0.001 km^2 or (**b**) from ref. (*78*) for lakes >0.1 km^2 within the same latitudinal range as our study sites, i.e. 37.9-64.3°N. We plotted the total number of lakes in $\log_{10}$ intervals at their corresponding midpoints.

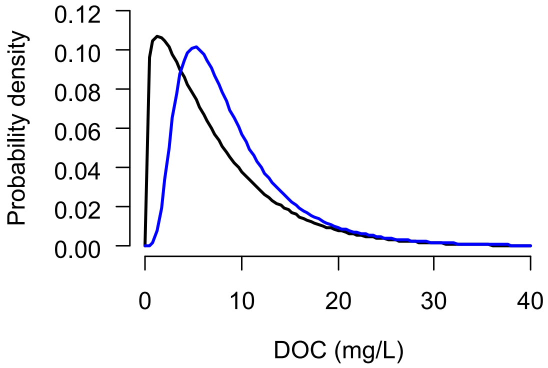

fig. S8. DOC distributions from 7514 worldwide lakes. Data from ref. (*51*) (black line) and 113 site × date observations in our dataset (blue line). The probability density for our dataset was estimated from a log-normal distribution, which was better supported than a Gamma distribution (ΔAIC = -10), which was used to derive the density function of Sobek et al. (*51*, eqn 1).

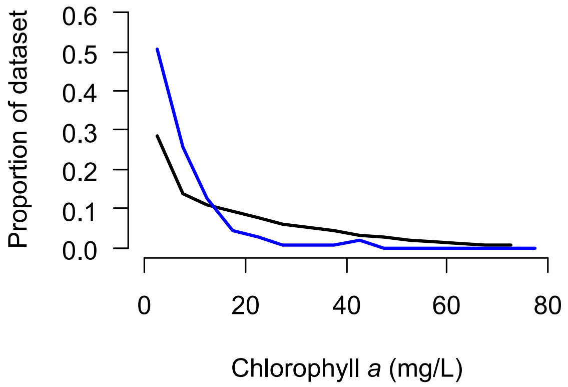

fig. S9. Chlorophyll *a* distribution from 80,012 worldwide lakes. Data from ref. (*52*) (black line) and 113 site × date observations in our dataset (blue line). We plotted the total number of lakes in 5 mg/L intervals at their corresponding midpoints.

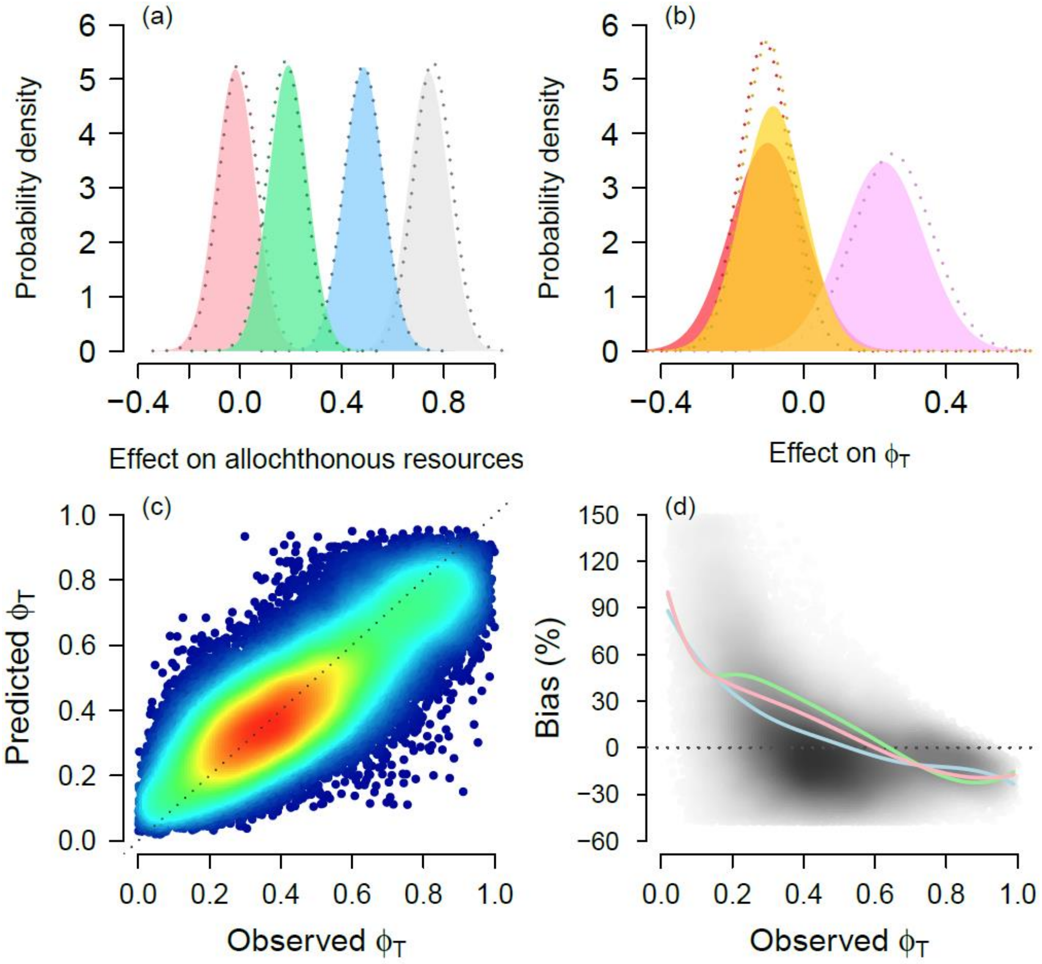

fig. S10. Model recovers known parameters across 100 simulated data sets that span the range of φ_T (that is, 0 to 1). Mean posterior distributions of the effects of: (**a**) DOC (gray), NDVI (pink), ratio of lake perimeter to area (blue), and area of woody vegetation per meter shoreline (green) on availability of allochthonous resources; (**b**) allochthonous resources (pink), lake chlorophyll *a* (gray), and their interaction (blue) on terrestrial resource use (φ_T); dashed lines are known prior distributions. (**c**) Mean predicted versus observed (i.e. known) φ_T for 559 consumer observations in each of 100 simulations. Warmer colours indicate greater concentration of points (total n = 55,900). (**d**) Percent bias in mean predicted φ_T values. Darker shading indicates greater concentration of points. Lines are splines fitted through observations upon one (δ^2H-only, pink), two (δ^{13}C-δ^{15}N, green), or three (δ^{13}C-δ^{15}N-δ^2H, blue) isotopes.

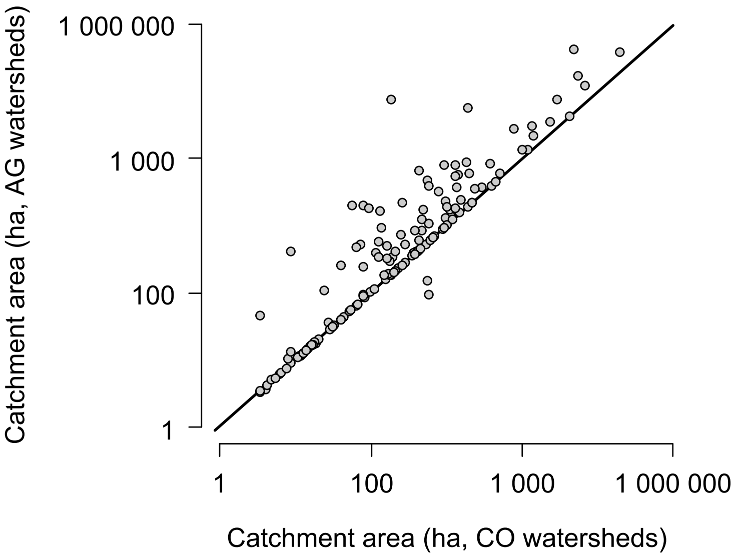

fig. S11. Catchment area estimated for 147 lakes in our isotope data set. Areas were estimated using either the aggregate (AG) or context (CO) catchment approach.

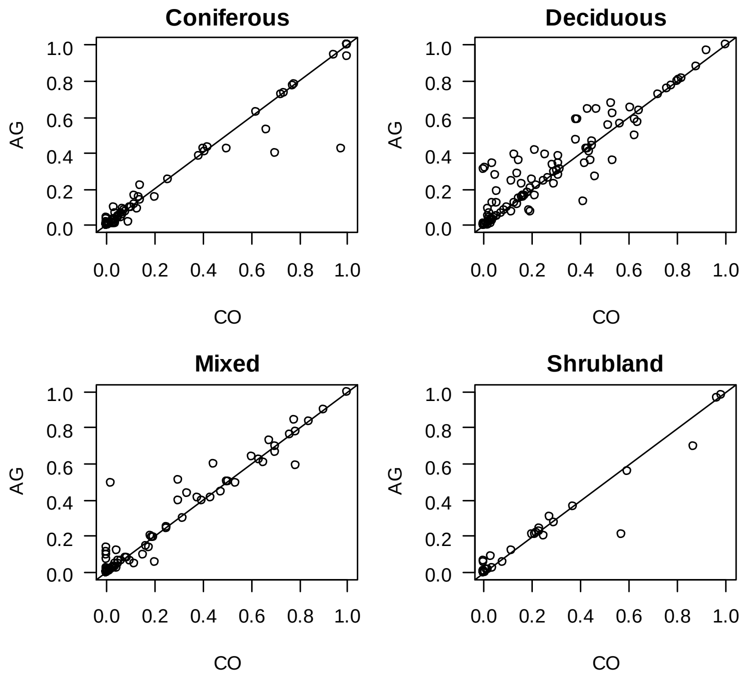

fig. S12. Proportion of each catchment covered with one of four woody vegetation types. Values estimated using either the aggregate (AG) or context (CO) approach. n = 147 lakes.

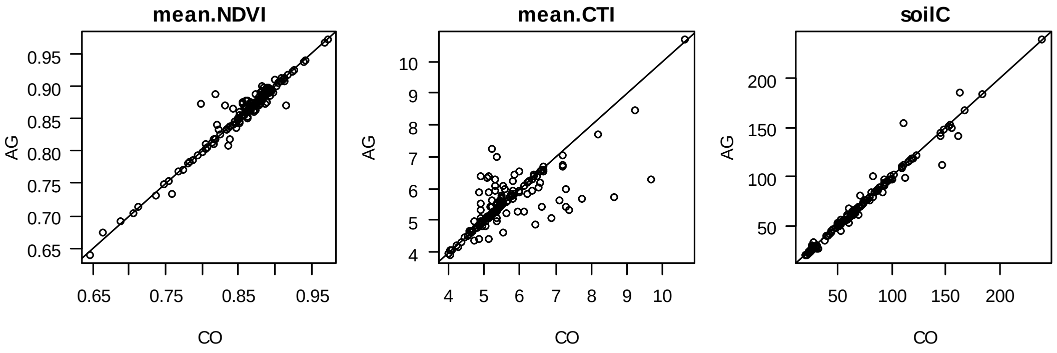

fig. S13. Vegetation, geomorphology, and soil characteristics. Catchments delineated with either the aggregate (AG) or context (CO) approach. *n* = 147 lakes.

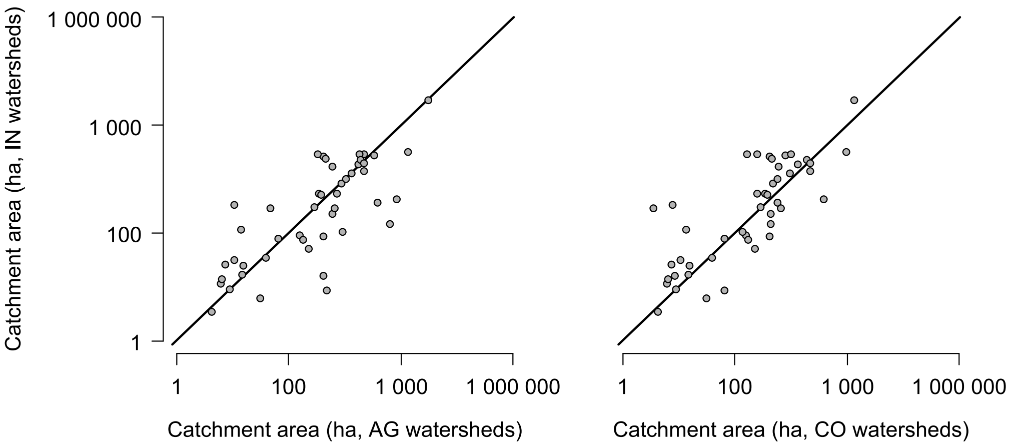

fig. S14. Catchment area for 46 lakes. Areas estimated using data provided by different investigators contributing stable isotope data. We compared the investigator estimates (IN) to those from the (**a**) aggregate (AG) and (**b**) context (CO) catchment approach.

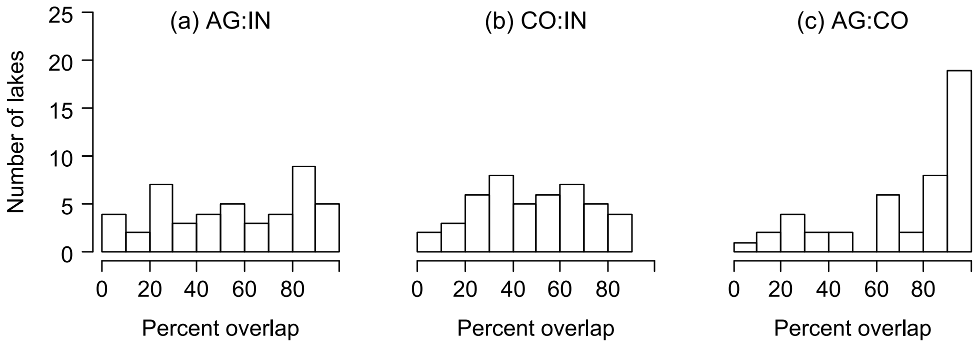

fig. S15. Percent overlap in catchments of each of the 46 lakes delineated with three different approaches. Catchments that we compared were the (**a**) aggregate (AG) and investigator (IN), (**b**) context (CO) and IN, and (**c**) AG and CO approaches.

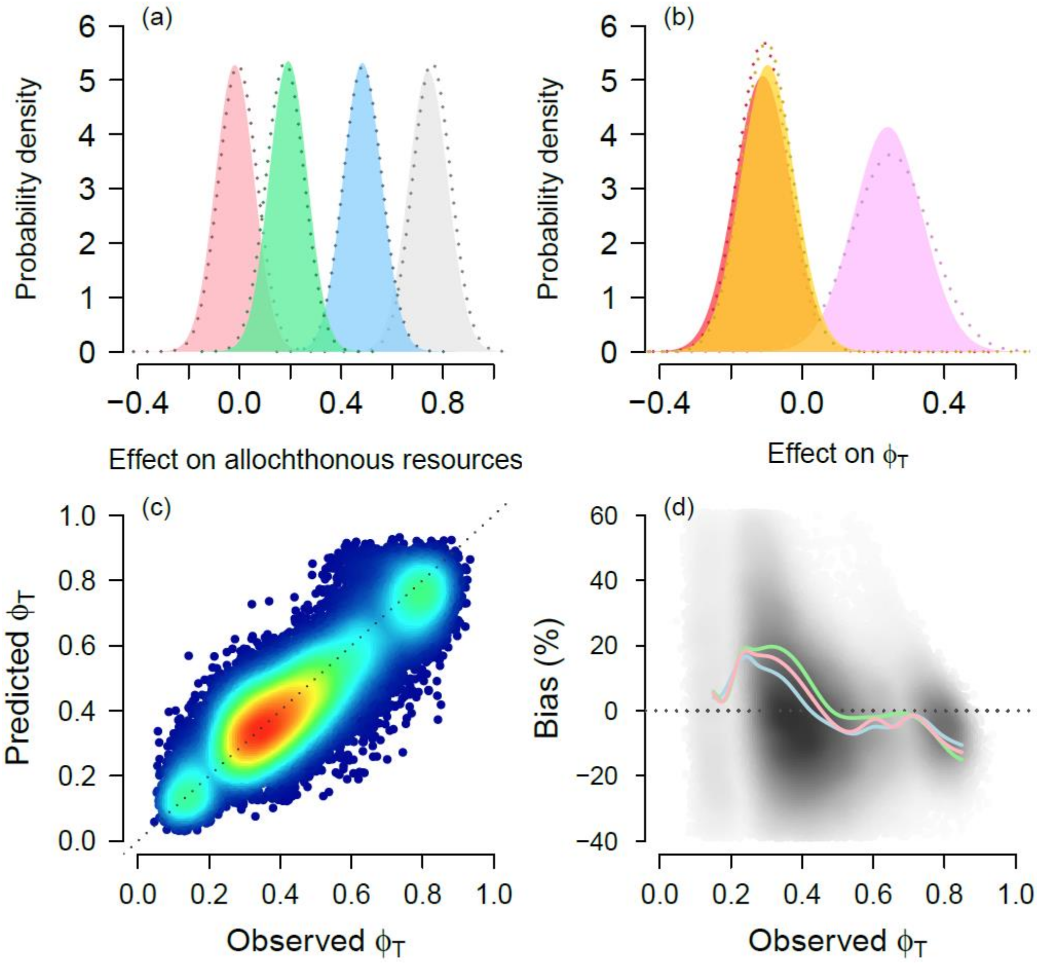

fig. S16. Model recovers known parameters despite random noise around the mean effects of covariates predicting the availability of allochthonous resources ξ_{kl}. Mean posterior distributions across 100 simulations of the effects of (**a**) DOC (gray), NDVI (pink), ratio of lake perimeter to area (blue), and area of woody vegetation per meter shoreline (green) on availability of allochthonous resources; (**b**) allochthonous resources (purple), lake chlorophyll *a* (red), and their interaction (orange) on terrestrial resource use (φ_T); dashed lines are known prior distributions. (**c**) Mean predicted versus observed (i.e. known) φ_T for 559 consumer observations in each of 100 simulations. Warmer colours indicate greater concentration of points (total n = 55,900). (**d**) Percent bias in mean predicted φ_T values. Darker shading indicates greater concentration of points and lines are splines fitted through observations upon one (δ^2H-only, pink), two (δ^{13}C-δ^{15}N, green), or three (δ^{13}C-δ^{15}N-δ^2H, blue) isotopes.

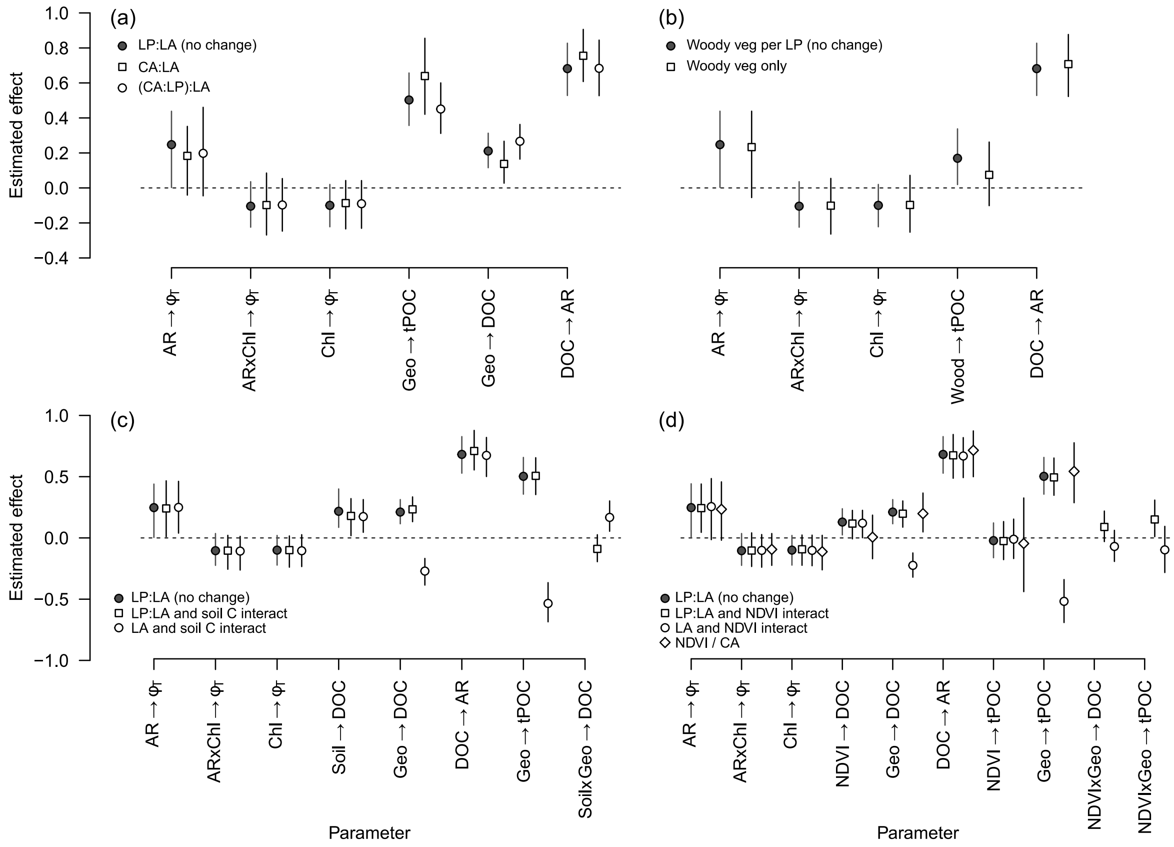

fig. S17. Alternate ways of modeling t-OM deposition. Points are mean ± 95% CIs for estimated effects of allochthonous resources (AR), chlorophyll *a* (Chl), geomorphology (Geo), woody vegetation in the surrounding catchment per meter shoreline (Wood), soil carbon (Soil), and NDVI on terrestrial resource support (ϕ_T), terrestrially derived POC, or DOC; arrows indicate effect direction. Geo was expressed as either the ratio of lake perimeter (LP) to lake area (LA), ratio of catchment area (CA) to LA, or simply as LA. Models were fitted as in the main text but replaced: (**a**) LP:LA with CA:LA, and (**b**) Wood with a measure that was not expressed relative to LP. We also expressed (**c**) Soil and (**d**) NDVI per LA and/or CA. To avoid multicollinearity with variables that already accounted for LA and CA, such as Wood and Geo, we included LA on its own in the model described by eqns S7 and S8 in methods S2 and allowed its effect to vary with either Soil or NDVI (i.e. statistical interaction). This parameterisation was analogous with expressing Soil and NDVI per unit lake area. The statistical interaction implied, for example in (c), that larger lakes had less DOC but that this was attenuated as the surrounding catchment had more soil carbon.

table S1. Mean and 95% CIs for model parameter estimates associated with eqs. S1 to S11. Bolded effects have 95% CIs that exclude zero.

Parameter	Mean	2.5%	97.5%
Mean ϕ_T α_1	-0.56	-1.36	0.12
Mean DOC α_3	2.03	1.92	2.12
Mean water colour α_5	0.69	0.56	0.82
Mean TN α_6	5.94	5.83	6.02
Mean chlorophyll *a* α_7	1.46	1.30	1.61
Effect of spring on ϕ_T $\alpha_{2[s=1]}$	1.19	-0.30	3.48
**Effect of autumn on ϕ_T $\alpha_{2[s=2]}$**	**0.57**	**0.35**	**0.77**
**Effect of winter on ϕ_T $\alpha_{2[s=3]}$**	**1.29**	**0.19**	**3.10**
**Effect of urban land on TN $\alpha_{6[k=1]}$**	**0.82**	**0.60**	**1.01**
**Effect of allochthonous resources on ϕ_T β_1**	**0.24**	**0.01**	**0.46**
Effect of chlorophyll *a* on ϕ_T β_2	-0.10	-0.25	0.02
Effect of chlorophyll *a* × allochthonous resources interaction on ϕ_T β_3	-0.10	-0.23	0.05
**Effect of DOC on allochthonous resources β_4**	**0.69**	**0.53**	**0.83**
**Effect of NDVI on DOC β_6**	**0.13**	**0.02**	**0.25**
**Effect of soil C on DOC β_7**	**0.22**	**0.08**	**0.36**
**Effect of soil wetness on DOC β_8**	**0.22**	**0.09**	**0.34**
**Effect of LP:LA on DOC β_9**	**0.22**	**0.10**	**0.33**
**Effect of air temperature on DOC β_{10}**	**0.14**	**<0.01**	**0.27**
Effect of NDVI on β_5POC β_{11}	-0.02	-0.17	0.13
**Effect of woody vegetation cover on β_5POC β_{12}**	**0.18**	**0.03**	**0.33**
Effect of soil wetness on β_5POC β_{13}	-0.01	-0.15	0.15
**Effect of LP:LA on β_5POC β_{14}**	**0.50**	**0.36**	**0.66**
**Effect of air temperature on β_5POC β_{15}**	**-0.34**	**-0.49**	**-0.19**
**Effect of allochthonous resources on TN β_{16}**	**0.28**	**0.20**	**0.36**
**Effect of colour on chlorophyll *a* β_{17}**	**-0.15**	**-0.28**	**-0.01**
**Effect of TN on chlorophyll *a* β_{18}**	**0.62**	**0.45**	**0.76**
**Effect of TP on chlorophyll *a* β_{19}**	**0.27**	**0.12**	**0.43**
**Effect of TN × TP interaction on chlorophyll *a* β_{20}**	**0.24**	**0.12**	**0.39**
Mean of missing water chemistry values υ_{gk}	0.23	-0.09	0.54

SD in $\phi_T \sigma_T$	0.04	0.02	0.06
SD of DOC	0.55	0.48	0.64
SD of POC	0.45	0.17	0.66
SD of water colour	0.55	0.28	0.72
SD of TN	0.44	0.38	0.51
SD of chlorophyll *a*	0.60	0.53	0.70
Consumer variation in mean ϕ_T	0.39	0.18	0.80
Consumer variation in effect of allochthonous resources on ϕ_T	0.18	0.02	0.44
Consumer variation in effect of chlorophyll *a* on ϕ_T	0.06	<0.01	0.24
Consumer variation in effect of chlorophyll *a* × allochthonous resources interaction on ϕ_T	0.10	0.01	0.32
SD of effect of research group variation on ϕ_T	0.85	0.49	1.56
SD of missing water chemistry values υ_{gk}	0.09	<0.01	0.34
SD of $\delta^{13}C$ in three-isotope model	2.40	2.12	2.72
SD of $\delta^{15}N$ in three-isotope model	1.28	1.13	1.46
SD of $\delta^{2}H$ in three-isotope model	9.30	7.30	11.3
SD of $\delta^{13}C$ in two-isotope model	4.32	3.80	4.92
SD of $\delta^{15}N$ in two-isotope model	2.21	1.96	2.56
SD of $\delta^{2}H$ in one-isotope model	4.50	1.00	7.00
Trophic position of *Bosmina*	0.98	0.81	1.16
Trophic position of bulk zooplankton	1.04	0.85	1.23
Trophic position of calanoids	1.26	1.07	1.44
Trophic position of *Chaoborus*	1.98	1.80	2.19
Trophic position of cladocera	0.87	0.70	1.06
Trophic position of copepods	1.29	1.11	1.48
Trophic position of cyclopoids	1.47	1.30	1.67
Trophic position of *Daphnia*	0.87	0.69	1.04
Trophic position of *Eudiaptomous*	1.28	1.11	1.49
Trophic position of *Holopedium*	0.89	0.71	1.07
Per-trophic-level fractionation of *Bosmina*	2.91	1.01	4.66
Per-trophic-level fractionation of bulk zooplankton	4.25	3.69	4.79
Per-trophic-level fractionation of calanoids	6.14	5.52	6.78
Per-trophic-level fractionation of *Chaoborus*	7.71	7.22	8.20

Per-trophic-level fractionation of cladocera	3.30	2.76	3.86
Per-trophic-level fractionation of copepods	5.94	5.15	6.81
Per-trophic-level fractionation of cyclopoids	5.98	5.31	6.75
Per-trophic-level fractionation of *Daphnia*	2.86	2.17	3.47
Per-trophic-level fractionation of *Eudiaptomous*	6.35	5.28	7.41
Per-trophic-level fractionation of *Holopedium*	2.87	1.91	3.95
Contribution of dietary water towards $\delta^{2}H$ of *Bosmina*	0.27	0.19	0.35
Contribution of dietary water towards $\delta^{2}H$ of bulk zooplankton	0.13	0.09	0.17
Contribution of dietary water towards $\delta^{2}H$ of calanoids	0.10	0.06	0.15
Contribution of dietary water towards $\delta^{2}H$ of *Chaoborus*	0.08	0.05	0.12
Contribution of dietary water towards $\delta^{2}H$ of cladocera	0.16	0.11	0.21
Contribution of dietary water towards $\delta^{2}H$ of copepods	0.14	0.09	0.18
Contribution of dietary water towards $\delta^{2}H$ of cyclopoids	0.18	0.13	0.23
Contribution of dietary water towards $\delta^{2}H$ of *Daphnia*	0.16	0.11	0.21
Contribution of dietary water towards $\delta^{2}H$ of *Eudiaptomous*	0.20	0.09	0.35
Contribution of dietary water towards $\delta^{2}H$ of *Holopedium*	0.17	0.12	0.23
Correlation between colour and TN	0.46	0.15	0.67
Correlation between $\delta^{13}C$ and $\delta^{15}N$ in three-isotope model	-0.26	-0.39	-0.10
Correlation between $\delta^{13}C$ and $\delta^{2}H$ in three-isotope model	0.30	0.13	0.43
Correlation between $\delta^{15}N$ and $\delta^{2}H$ in three-isotope model	-0.02	-0.19	0.15
Correlation between $\delta^{13}C$ and $\delta^{15}N$ in two-isotope model	0.19	-0.01	0.37

table S2. Key symbols and abbreviations used in the text and the Supplementary Materials and Methods.

Abbreviation	Definition
CA	Catchment area
CTI	Compound Topographic Index that approximates soil wetness
g	Index for research group
i	Index for individual observations
j	Index for consumer type
k	Index for study site
LA	Lake area
LP	Lake perimeter
LPLA	Ratio of LP to LA
MOB	Methane oxidising bacteria
NDVI	Normalized Difference Vegetation Index that approximates vegetation density
s	Index for season
Soil	Mean catchment soil carbon
Temp	Mean daily air temperature during warmest quarter from 1950 to 2000
t-OM	Terrestrial organic matter
Wetness/WETS	Catchment wetness measured as mean CTI
Wood	Woody vegetation in surrounding catchment per meter shoreline
β_5POC	Total terrestrially derived POC
Δ	Trophic enrichment of $\delta^{15}N$
ξ	Availability of allochthonous resources
σ_O	Residual error associated with individual consumer isotope observations
σ_P	Residual error associated with pelagic end member observations
σ_{res}	Residual error associated with ϕ_T
σ_T	Residual error associated with terrestrial end member observations
σ_W	Residual error associated with environmental water samples

σ_τ	Residual error in trophic position of each consumer
σ_ω	Residual error in per-trophic-level contribution of dietary water to δ^2H
τ	Trophic levels above primary producers
ϕ_P	Proportional use of pelagic resources
ϕ_T	Proportional use of terrestrial resources
ω	Proportion of δ^2H obtained from environmental water
ω_P	Per-trophic-level contribution of environmental water to consumer isotope ratios

table S3. Reclassification of 2005 North America Land Cover. Classes were provided by the Commission for Environmental Cooperation (*70*), to which we then assigned a new classification.

Value	Class	New classification
1	Temperate or sub-polar needleleaf forest	Coniferous Forest
2	Sub-polar taiga needleleaf forest	Coniferous Forest
3	Tropical or sub-tropical broadleaf evergreen forest	Coniferous Forest
4	Tropical or sub-tropical broadleaf deciduous forest	Deciduous Forest
5	Temperate or sub-polar broadleaf deciduous forest	Deciduous Forest
6	Mixed forest	Mixed Forest
7	Tropical or sub-tropical shrubland	Shrubland
8	Temperate or sub-polar shrubland	Shrubland
9	Tropical or sub-tropical grassland	Grassland
10	Temperate or sub-polar grassland	Grassland
11	Sub-polar or polar shrubland-lichen-moss	Sparse Vegetation
12	Sub-polar or polar grassland-lichen-moss	Sparse Vegetation
13	Sub-polar or polar barren-lichen-moss	Sparse Vegetation
14	Wetland	Wetland
15	Cropland	Cropland
16	Barren lands	Barren
17	Urban	Urban
18	Water	Water
19	Snow	Snow

table S4. Reclassification of 2006 European Land Cover. Classes (denoted by LABEL1, LABEL2, and LABEL 3, which correspond with increasing specificity) were provided by the European Environment Agency (*71*), to which we then assigned a new classification.

Value (Original EEA table: GRD_CODE)	LABEL1	LABEL2	LABEL3	New classification
1	Artificial surfaces	Urban fabric	Continuous urban fabric	Urban
2	Artificial surfaces	Urban fabric	Discontinuous urban fabric	Urban
3	Artificial surfaces	Industrial, commercial and transport units	Industrial or commercial units	Urban
4	Artificial surfaces	Industrial, commercial and transport units	Road and rail networks and associated land	Urban
5	Artificial surfaces	Industrial, commercial and transport units	Port areas	Urban
6	Artificial surfaces	Industrial, commercial and transport units	Airports	Urban
7	Artificial surfaces	Mine, dump and construction sites	Mineral extraction sites	Urban
8	Artificial surfaces	Mine, dump and construction sites	Dump sites	Urban
9	Artificial surfaces	Mine, dump and construction sites	Construction sites	Urban
10	Artificial surfaces	Artificial, non-agricultural vegetated areas	Green urban areas	Urban
11	Artificial surfaces	Artificial, non-agricultural vegetated areas	Sport and leisure facilities	Urban
12	Agricultural areas	Arable land	Non-irrigated arable land	Cropland
13	Agricultural areas	Arable land	Permanently irrigated land	Cropland
14	Agricultural areas	Arable land	Rice fields	Cropland

15	Agricultural areas	Permanent crops	Vineyards	Cropland
16	Agricultural areas	Permanent crops	Fruit trees and berry plantations	Cropland
17	Agricultural areas	Permanent crops	Olive groves	Cropland
18	Agricultural areas	Pastures	Pastures	Grassland
19	Agricultural areas	Heterogeneous agricultural areas	Annual crops associated with permanent crops	Cropland
20	Agricultural areas	Heterogeneous agricultural areas	Complex cultivation patterns	Cropland
21	Agricultural areas	Heterogeneous agricultural areas	Land principally occupied by agriculture, with significant areas of natural vegetation	Cropland
22	Agricultural areas	Heterogeneous agricultural areas	Agro-forestry areas	Cropland
23	Forest and semi natural areas	Forests	Broad-leaved forest	Deciduous Forest
24	Forest and semi natural areas	Forests	Coniferous forest	Coniferous Forest
25	Forest and semi natural areas	Forests	Mixed forest	Mixed Forest
26	Forest and semi natural areas	Scrub and/or herbaceous vegetation associations	Natural grasslands	Grassland
27	Forest and semi natural areas	Scrub and/or herbaceous vegetation associations	Moors and heathland	Shrubland
28	Forest and semi natural areas	Scrub and/or herbaceous vegetation associations	Sclerophyllous vegetation	Shrubland
29	Forest and semi natural areas	Scrub and/or herbaceous vegetation associations	Transitional woodland-shrub	Shrubland

30	Forest and semi natural areas	Open spaces with little or no vegetation	Beaches, dunes, sands	Barren
31	Forest and semi natural areas	Open spaces with little or no vegetation	Bare rocks	Barren
32	Forest and semi natural areas	Open spaces with little or no vegetation	Sparsely vegetated areas	Sparse Vegetation
33	Forest and semi natural areas	Open spaces with little or no vegetation	Burnt areas	Barren
34	Forest and semi natural areas	Open spaces with little or no vegetation	Glaciers and perpetual snow	Snow
35	Wetlands	Inland wetlands	Inland marshes	Wetland
36	Wetlands	Inland wetlands	Peat bogs	Wetland
37	Wetlands	Maritime wetlands	Salt marshes	Wetland
38	Wetlands	Maritime wetlands	Salines	Wetland
39	Wetlands	Maritime wetlands	Intertidal flats	Wetland
40	Water bodies	Inland waters	Water courses	Water
41	Water bodies	Inland waters	Water bodies	Water
42	Water bodies	Marine waters	Coastal lagoons	Water
43	Water bodies	Marine waters	Estuaries	Water
44	Water bodies	Marine waters	Sea and ocean	Water

table S5. Consumer-specific dietary parameters. Mean trophic levels above primary production τ varied among consumers with a SD of 0.1 for all (*7*, *28*). Mean and SD of per-trophic-level contribution of water to consumer isotope ratios ω_P was derived from averaging multiple values in ref. (*42*); we used values from higher taxonomic levels where data were missing for specific consumers.

Consumer	Mean τ	Mean ω_P	SD ω_P
Bosmina	1.00	0.290	0.040
Bulk zooplankton	1.15	0.245	0.040
Calanoida	1.25	0.160	0.050
Chaoborus	2.00	0.135	0.045
Cladocera	1.00	0.290	0.040
Copepoda	1.30	0.160	0.040
Cyclopoida	1.50	0.160	0.030
Daphnia	1.00	0.290	0.040
Eudiaptomus	1.25	0.160	0.050
Holopedium	1.00	0.290	0.040
